# Comments and Illustrations of Ultrasound Findings in Extrapulmonary Tuberculosis Manifestations

**DOI:** 10.3390/diagnostics14070706

**Published:** 2024-03-27

**Authors:** Kathleen Möller, Axel Löwe, Christian Jenssen, Nitin Chaubal, Heike Gottschall, Benjamin Misselwitz, Meghana Reddy Kurapati, Anoop Reddy Puritipati, Yi Dong, Siegbert Faiss, Christoph F. Dietrich

**Affiliations:** 1 Medical Department I/Gastroenterology, Sana Hospital Lichtenberg, 10365 Berlin, Germany; k.moeller@live.de (K.M.); heike.gottschall@sana.de (H.G.); siegbert.faiss@sana.de (S.F.); 2 Department Allgemeine Innere Medizin (DAIM), Kliniken Hirslanden Beau Site, Salem und Permanence, 3013 Bern, Switzerland; axel.loewe@hirslanden.ch; 3 Department of Internal Medicine, Krankenhaus Märkisch-Oderland, 15344 Strausberg, Germany; c.jenssen@khmol.de (C.J.); anoopreddy27@gmail.com (A.R.P.); 4 Brandenburg Institute for Clinical Ultrasound (BICUS), Medical University Brandenburg, 16816 Neuruppin, Germany; 5 Thane Ultrasound Center, Thane 400601, India; thaneultrasound@gmail.com; 6 Jaslok Hospital & Research Centre, Mumbai 400026, India; 7 Inselspital, University of Bern, 3010 Bern, Switzerland; benjamin.misselwitz@insel.ch; 8 Department of Ultrasound, Xinhua Hospital, Shanghai Jiao Tong University School of Medicine, Shanghai 200011, China; drdaisydong@hotmail.com

**Keywords:** tuberculosis, extrapulmonary manifestations, ultrasonography, contrast enhanced ultrasonography, endoscopic ultrasound

## Abstract

This review describes the appearance of extrapulmonary tuberculosis manifestations in comprehensive and multiparametric ultrasound imaging. The aim is to increase awareness of typical ultrasound findings regarding extrapulmonary tuberculosis, correlate those with pathological features, and facilitate differential diagnosis. Point of care ultrasound protocols can be used as a screening method in high-risk populations, although the negative findings do not exclude tuberculosis. Conversely, the diagnosis of extrapulmonary tuberculosis can never be made using ultrasound alone, as many ultrasound findings in extrapulmonary tuberculosis are non-specific. However, ultrasound-based sampling techniques can significantly facilitate the collection of samples for microbiological or molecular proof of tuberculosis, as well as facilitating the establishment of alternative diagnoses.

## 1. Introduction

Globally, tuberculosis (TB) is one of the leading causes of death [1]. In 2022, an estimated 10.6 million people worldwide contracted tuberculosis. HIV-infected people accounted for 6.3% of the total number, with the disease causing 1.30 million deaths of whom 167,000 people also had HIV. Globally, there is still a large gap between the estimated number of people with tuberculosis and the number of newly diagnosed people. In 2022, it was estimated that around 3.1 million people were not appropriately diagnosed with the disease or were not officially reported to the national authorities [2]. Typically, tuberculosis is localized in the lung, but 11–20% of patients have extrapulmonary manifestations [3,4]. Lymph nodes or pleura are usually affected. However, almost all organ systems can be involved. The extrapulmonary manifestations can occur in both immunocompetent and immunocompromised patients, but the simultaneous presence of an HIV infection significantly increases the probability. HIV patients are at risk of reactivating a tuberculosis infection and are more likely to develop disseminated disease [5,6,7,8]. The disease is endemic in areas with difficult socio-economic standards. In 2022, according to data from the WHO, most new cases of tuberculosis occurred in the Southeast Asian region (46%), then followed by the African region (23%) and the Western Pacific region (18%). Around 87% of new TB cases were concentrated in 30 countries (half of the global population) with a high TB burden; more than two thirds of global cases occurred in only the eight following countries: India, Indonesia, China, the Philippines, Pakistan, Nigeria, Bangladesh, and the Democratic Republic of Congo [2]. Special ultrasound protocols have been developed to improve the diagnosis of extrapulmonary tuberculosis. FASH (Focused Assessment with Sonography for HIV and Tuberculosis) is an ultrasound protocol, designed to detect signs of extrapulmonary tuberculosis, including pleural and pericardial effusion, enlarged intra-abdominal lymph nodes, splenic and liver abscesses, and ascites [8,9,10,11,12,13]. Extended FASH (eFASH) incorporates an ultrasound examination of the chest, inferior lymph node stations, and the width of the inferior vena cava as an indication of right heart failure [14,15]. Ultrasound is a simple and widely used cost-effective examination method for the detection and differentiation of extrapulmonary manifestations, and is also available and applicable in economically disadvantaged regions of the world.

Typical pathology in tuberculosis are granulomatous inflammation, granuloma formation, caseous necrosis and abscess formation, fibrosis, and calcification. There are correlating ultrasound images for these morphological changes in all the affected organs (Table 1 and Table 2).

With the help of contrast-enhanced ultrasound (CEUS), organ lesions can be characterized and differentiated from other differential diagnoses. This requires additional specialized knowledge. This review provides a comprehensive overview into the topic of extrapulmonary tuberculosis ultrasound imaging and the useful application of CEUS.

## 2. Lung Parenchyma

We will introduce this review with a brief overview of ultrasound findings regarding pulmonary tuberculosis, as pulmonary manifestations should always be examined in the context of extraintestinal tuberculosis. For more comprehensive data, we refer the reader to the relevant literature. The most commonly reported parenchymal ultrasound findings are subpleural nodules and lung consolidation. The detection rate for tuberculous caverns was low. Subpleural nodules and lung consolidation can only be seen via the use of ultrasound if they are directly adjacent to the chest wall, and are not overlaid by the lung tissue with corresponding artifacts [16,17,18,19,20].

In a systematic review, the sensitivity of ultrasound in the diagnosis of subpleural nodules was 72–100%; for lung consolidation, this was 46–80%; and for the detection of caverns, this was only 4–30% [17]. The corresponding specificities for subpleural nodule, lung consolidation, and cavitation were 66.7%, 25.3%, and 89.3%, respectively, based on data from only one study [21] from this meta-analysis [17]. Ultrasound cannot be used to make a differential diagnosis between fibrosis, necrosis, and malignant processes. However, ultrasound is being considered for the monitoring of the response of lung consolidation in children and pregnant women undergoing tuberculostatic therapy [16,19]. It is not possible to distinguish between active and inactive pulmonary tuberculosis using ultrasound [20]. Ultrasound can also be used as part of an ultrasound-guided biopsy to obtain histology from subpleural lesions [22,23]. CEUS can be used to differentiate these lesions—solid tissue, necrosis, abscesses, encapsulated effusions, and malignant lesions can be differentiated [22].

## 3. Pleural Effusion

Pleural effusion can develop as a consequence of pulmonary tuberculosis [24]. About 3% to 25% of patients with tuberculosis have pleurisy [25]. In a study that included HIV patients, examining them using the eFASH protocol, pleural effusion was the most common finding (61%) [14]. Pleural effusion in tuberculosis is more often unilateral and right sided. In the case of a newly diagnosed pleural effusion, especially in tuberculosis endemic areas, the possibility of tuberculosis should always be considered. The effusion can persist over a long period of time, and can occur unilaterally. The effusion can form septations due to fibrin strands, and can lead to pleural thickening and calcification. Pleural effusion is usually anechoic. If it is hemorrhagic or infected and rich in protein, the effusion is echogenic. In the course of tuberculosis, echogenic strands and complex septations may occur [16,26,27].

In tuberculosis, pleural effusion, pleural thickening, and pleural calcification are of interest for diagnosis. When compared to chest CT, ultrasound can detect the effusion and pleural calcification very well. CT was more sensitive in detecting parietal pleural thickening. The assessment of pleural thickness via ultrasound requires experience and training, as well as the consequent use of high-frequency transducers. While the sensitivity of ultrasound for the diagnosis of pleural effusion (78%) and pleural calcifications (74%) was comparable to that of CT (71.8% and 72.6%) in a large retrospective study, it was significantly inferior in the detection of pleural thickening, at only 2.8% versus 21.8% when using CT. For the diagnosis of tuberculous pleurisy, the sensitivity of the B-mode ultrasound was 85.5%, the specificity was 72.3%, the diagnostic agreement rate was 60.2% and the positive predictive value was 83.4% [26].

The ultrasound-guided aspiration of pleural effusion with the evaluation of the lymphocyte count and the adenosine deaminase (ADA) level within the pleural fluid can support the diagnosis of tuberculous pleurisy [27]. Under ultrasound guidance, targeted biopsies can be taken from the pleura and examined for granulomas [28]. The Xpert MTB (Mycobacterium tuberculosis)/RIF test increases the diagnostic sensitivity for tuberculosis in histologic pleural samples [22]. The test was approved by the World Health Organization in 2013 for the rapid diagnosis of extrapulmonary tuberculosis [29]. Xpert MTB/RIF(Xpert) is a fully automated quantitative real-time polymerase chain reaction (PCR) test, used as a frontline test for tuberculosis in countries with high rates of tuberculosis. The Xpert test has differing levels of variable sensitivity in different samples of extrapulmonary manifestations. Compared to pleural effusion, biopsy material proved to be significantly more effective for detecting tuberculosis in both Xpert and Mycobacterium tuberculosis cultures. The sensitivity of Xpert and tuberculosis cultures in biopsy specimens were 69.6% and 16.4%, in comparison to the sensitivity of Xpert and tuberculosis cultures in pleural effusions, which were only 13.8% and 7.6% [22]. Using CEUS to differentiate between vital and necrotic tissue increased the diagnostic yield and the sensitivity of the Xpert MTB/RIF test. The highest sensitivity (73%) was achieved in specimens from nonenhanced necrotic tissue, identified using CEUS [22]. Xpert was positive in only 17% of non-necrotic pleural thickenings with homogeneous CEUS enhancement [22]. While CEUS is used for tumors to obtain ultrasound-guided biopsies from non-necrotic tumor parts, in the case of tuberculosis diagnostics, a positive Xpert test was more often obtained from necrotic areas.

## 4. Peritoneum

Peritoneal tuberculosis is the most common abdominal manifestation [3]. It includes peritoneum, peritoneal cavity, mesentery, and omentum. Peritoneal tuberculosis usually arises from the rupturing of necrotic tuberculous lymph nodes, and is often combined with other extrapulmonary abdominal manifestations [5,30], but also from hematogenous or lymphatic seeding [31]. There are different forms, including wet, fibrotic, dry, and sclerosing types [3,30,32]. Frequently, there is evidence of ascites [31,33], which is usually rich in proteins and cells, meaning the fluid cannot be anechoic. A rarer variant is the fibrotic-fixed type, with thickened peritoneum and mesentery and local encapsulated ascites. Another variant is dry peritoneal tuberculosis with nodular lesions in the mesentery and adhesions [30,32,34]. Last but not least, there is a sclerosing encapsulating form (abdominal “cocoon”) with an intestinal obstruction [3,32,34]. A “cerebral fissure sign” is usually a sign of greater omentum, indicating tuberculous peritonitis. With high-frequency transducers, the hyperechoic and thickened omentum is interlaced with irregular hypoechoic or heterogeneous striations. The authors called this ultrasound image, according to brain fissures, the “cerebral fissure sign”. This change was observed exclusively in tuberculosis, and not in peritoneal carcinomatosis [35].

**Figure 1:**
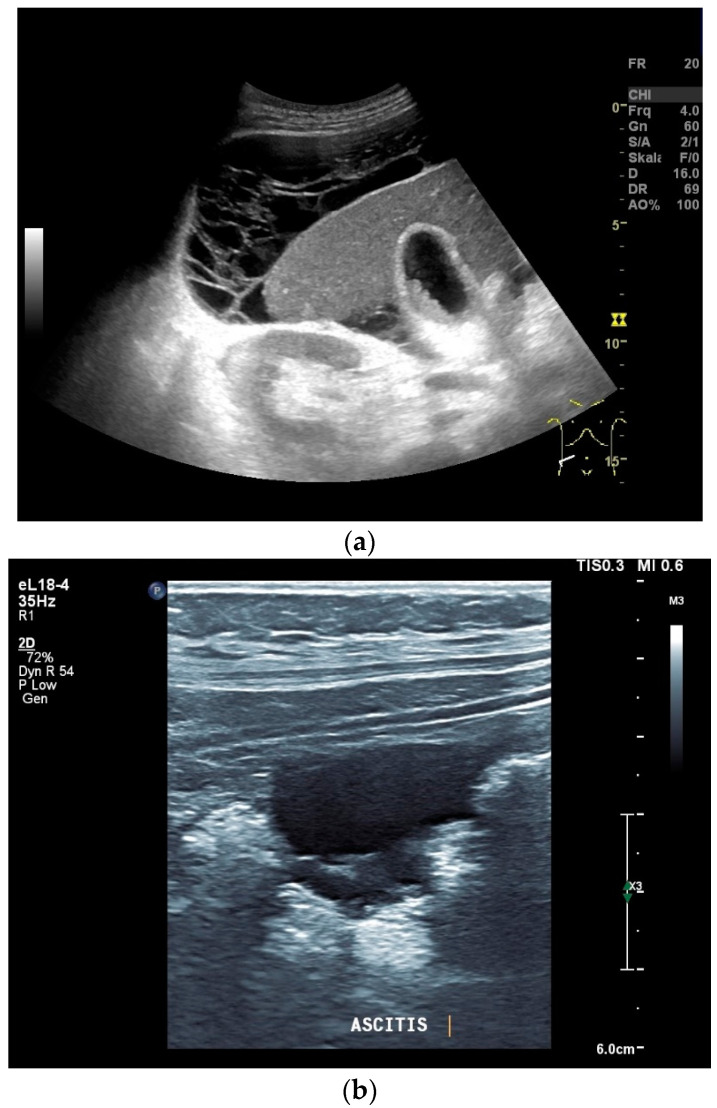

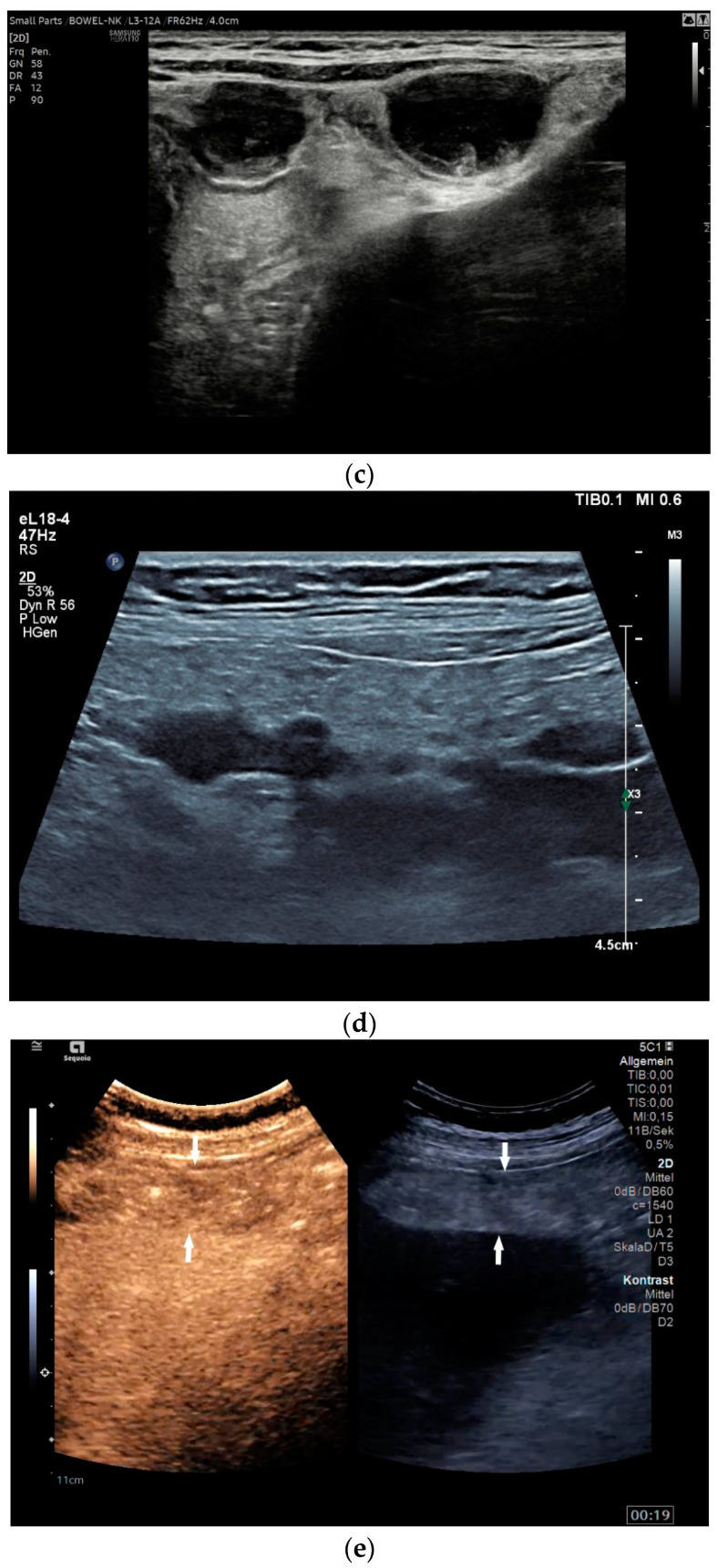

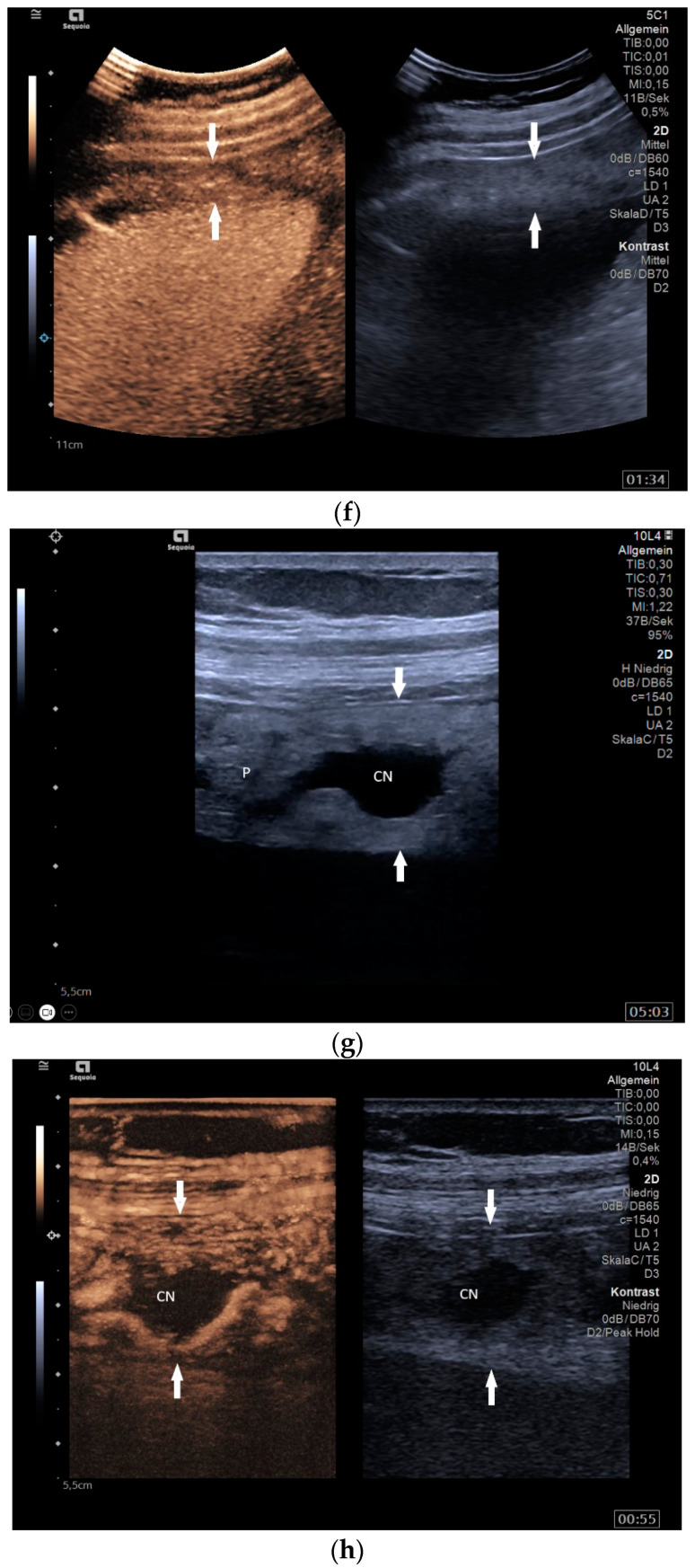
Typical changes in the peritoneum, mesentery, and omentum in patients with tuberculosis. Ascites with pronounced septa (**a**). Ascites encapsulated between the abdominal wall and colon (**b**). Omental thickening with non-echoic caseous abscesses and few echogenic contents (**c**); hypoechoic nodules in hyperechoic thickened peritoneum (**d**). Significantly thickened hyperechoic peritoneum in B-mode US (right side of image) (marked with arrows). In CEUS, the thickened peritoneum is contrast enhanced in the arterial phase (**e**). In the parenchymal phase, it shows a decrease in enhancement (**f**). The thickened hyperechoic peritoneum shows a non-echoic lesion (arrow) (**g**). In the CEUS, this is not enhanced and instead shows a hyperenhanced rim. This corresponds to caseous necrosis (**h**).

What can be seen sonographically? Ascites can be either diffuse or focal type [Figure 1a,b]. Due to the protein and cell content, these tend to be echogenic, rather than non-echoic. Attention must be paid to enlarged lymph nodes at the same time. Any thickening of the peritoneum and mesentery must be specifically searched for. This also applies to macronodular changes in the peritoneum and mesentery [Figure 1c–h]. The smallest speck-like deposits are usually not visible on ultrasound [36]. With ascites, it may be possible to observe the adhesions [31,37]. Kumar et al. were able to histologically detect granulomatous inflammation in 17/19 (89%) patients using ultrasound-guided needle biopsy of omental thickenings. In a country like India, the authors interpreted this as a manifestation of tuberculosis [32].

The most important differential diagnoses are ascites of other origins, peritonitis in the case of septations, and peritoneal carcinomatosis in the case mesentery and peritoneum, pseudomyxoma peritonei, and mesothelioma thickening. Diagnosis is challenging. Diagnostic laparoscopies and biopsies are the diagnostic gold standard if the diagnosis cannot be otherwise confirmed [31,37].

## 5. Lymph Nodes

Tuberculous lymphadenopathy is a frequent and typical manifestation. It accounts for about 35% of all extrapulmonary manifestations [38]. Tuberculous lymph nodes are predominantly described as enlarged and with a hypoechoic center. This corresponds to caseous necrosis. Strong internal echo and thin internal layers are reported. The lymph node hilus is usually destroyed by the necrosis [39,40]. When using endobronchial ultrasound (EBUS), heterogeneous echotexture or coagulation necrosis in the lymph nodes were specific findings for tuberculosis [41].

When using multiparametric ultrasound, the majority of superficial lymph nodes showed marginal blood flow during Color Doppler Imaging. When using CEUS, marginal/annular enhancement, separation enhancement, and heterogeneous enhancement was observed [42]. When using elastography, either type 1—Red or yellow-green or type 2—Blue < 45% have been described. However, elastography type 3 was also a typical finding in another endoscopic ultrasound (EUS) study [43]. It is conceivable that the elasticity depends on the stage of the disease, the presence of necrosis, or the development of fibrosis. Histologically evaluable material can be obtained from superficial lymph nodes or abdominal lymph nodes, accessible via percutaneous ultrasound-guided biopsy. The aim is to detect granulomatous inflammation, epithelioid cell granulomas, Langerhans cells, caseous necrosis, and the presence of Mycobacterium tuberculosis. Enlarged cervical lymph nodes are easily accessible for ultrasound-guided biopsy. The sensitivity and specificity of histologic diagnosis were 70.1% and 100%, respectively. The sensitivity and specificity of Xpert for cervical tuberculous lymphadenitis diagnoses were 82.5% and 97.5%, respectively [44]. The acquisition of histologically evaluable material can be improved by differentiating the lymph node structure in CEUS. Solid vital tissue can be differentiated from areas of necrosis. The CEUS-guided biopsy of lymph node tuberculosis has a high sampling success rate of 94.5% [44].

**Figure 2:**
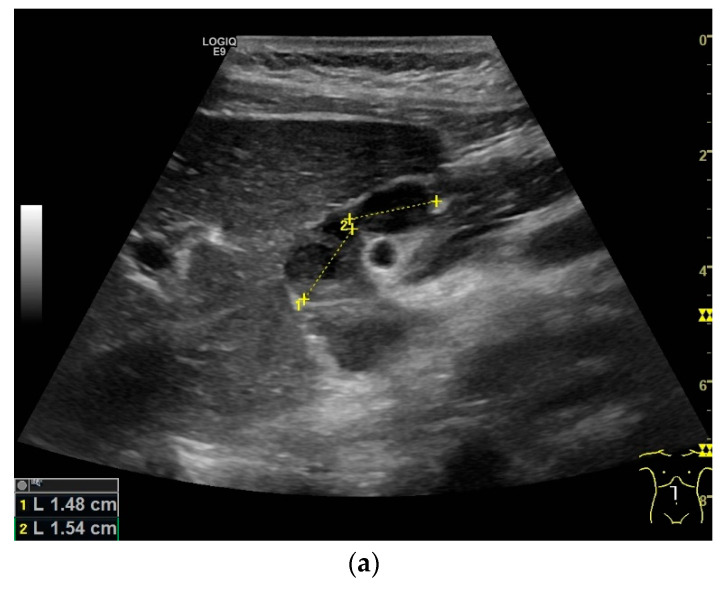

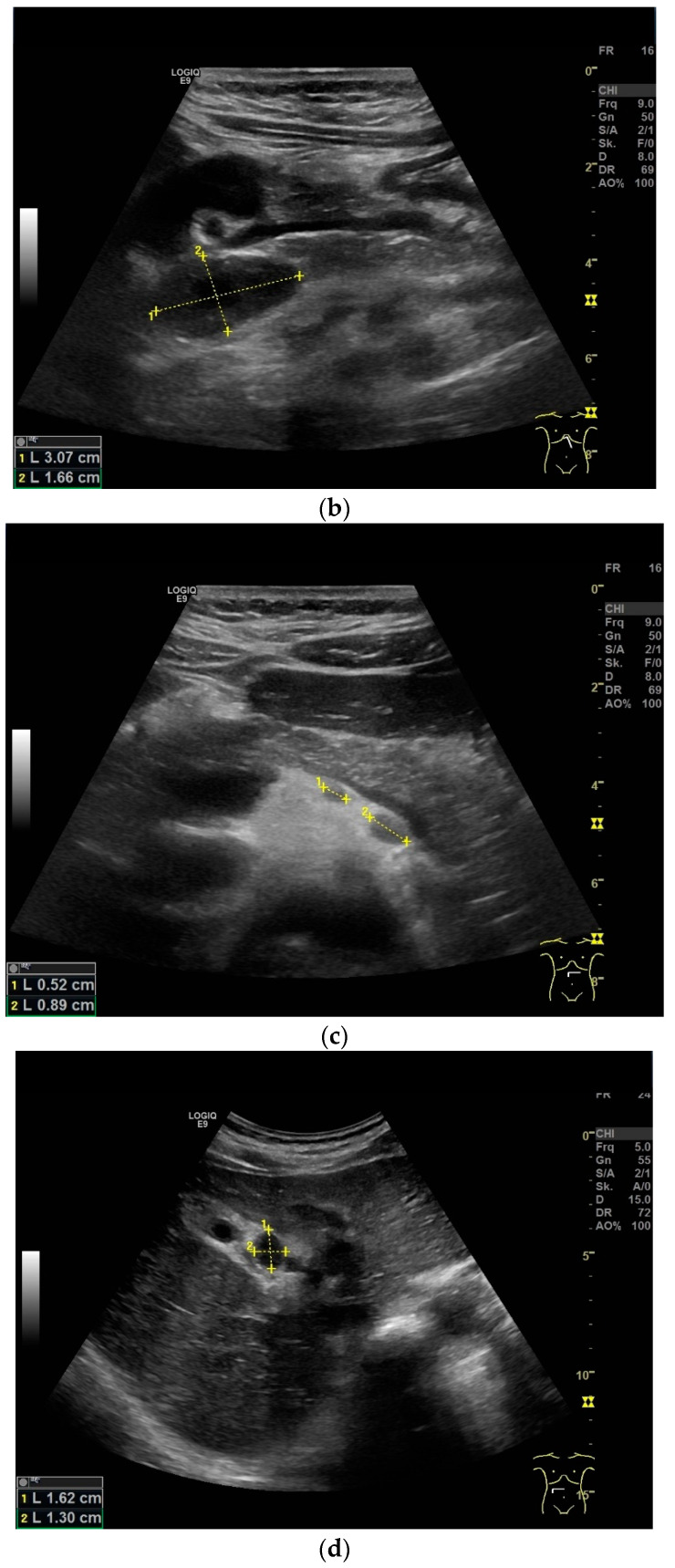

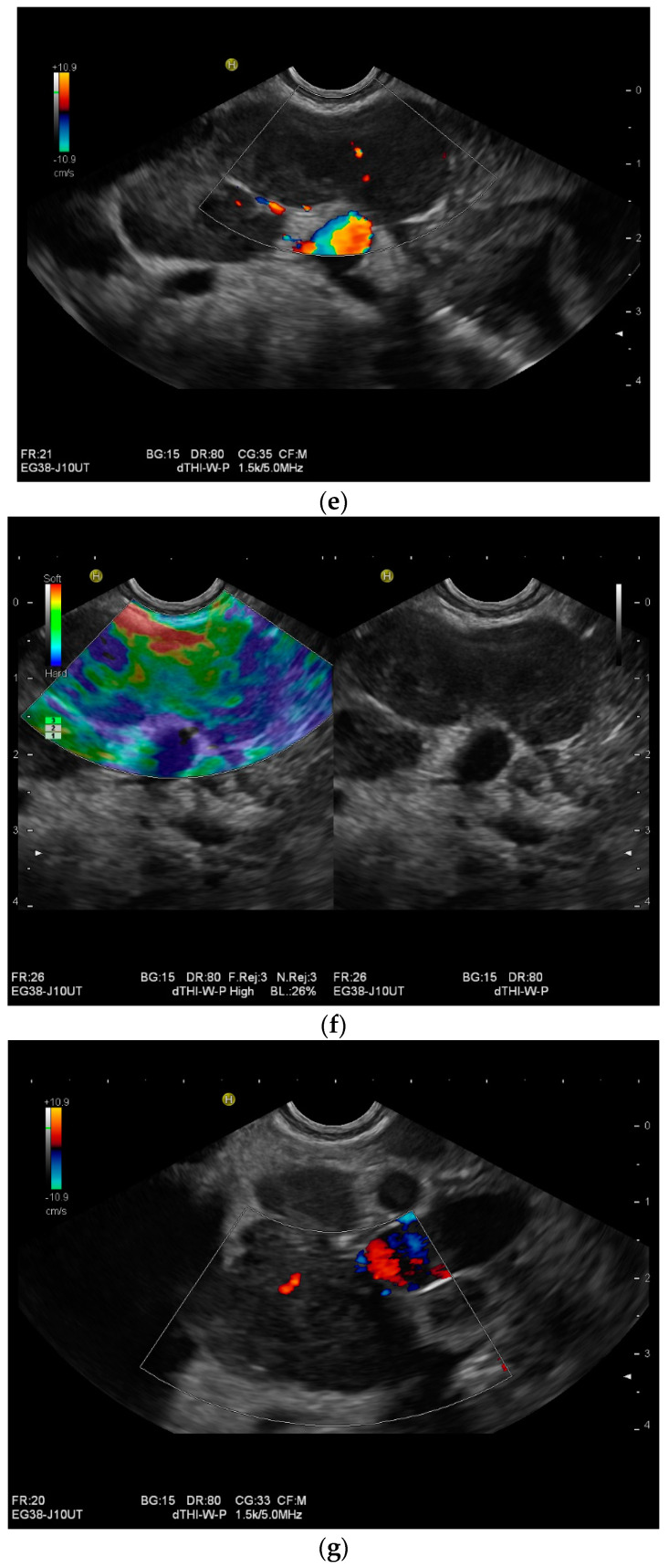

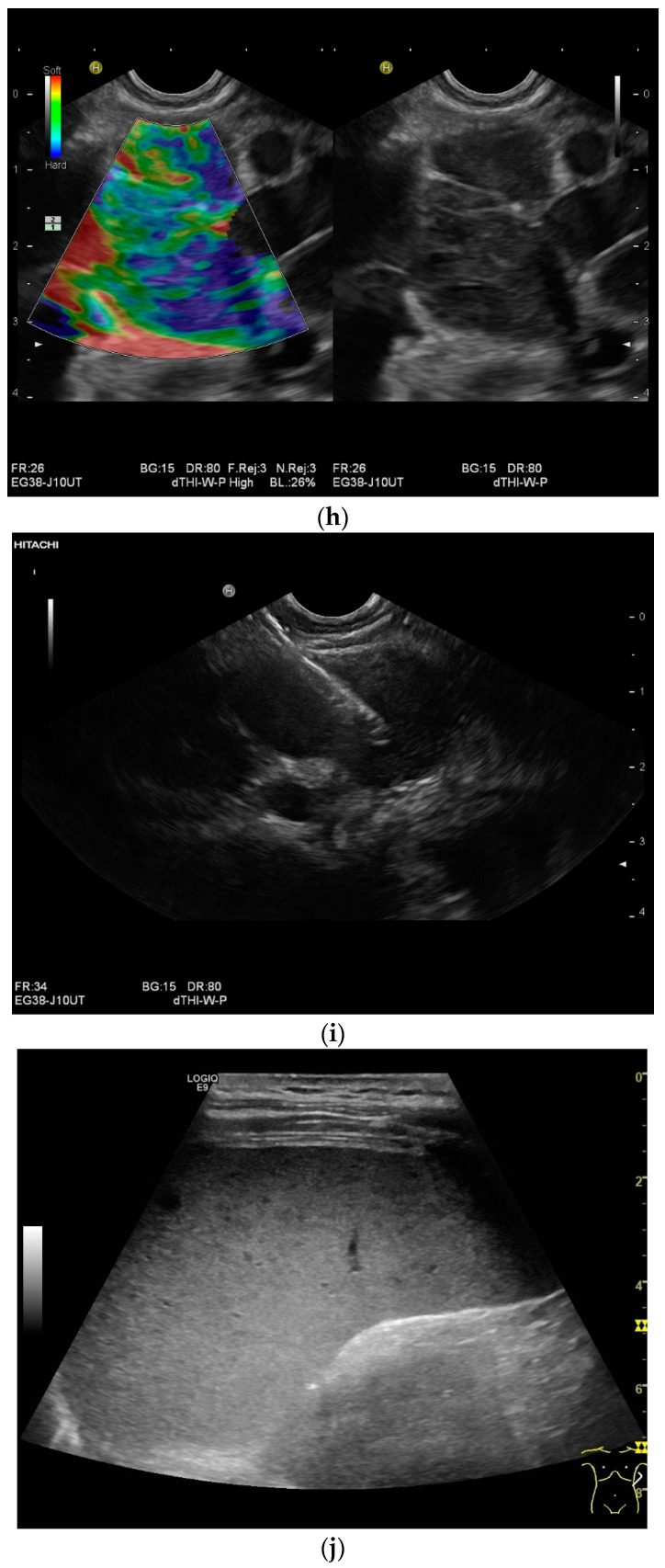

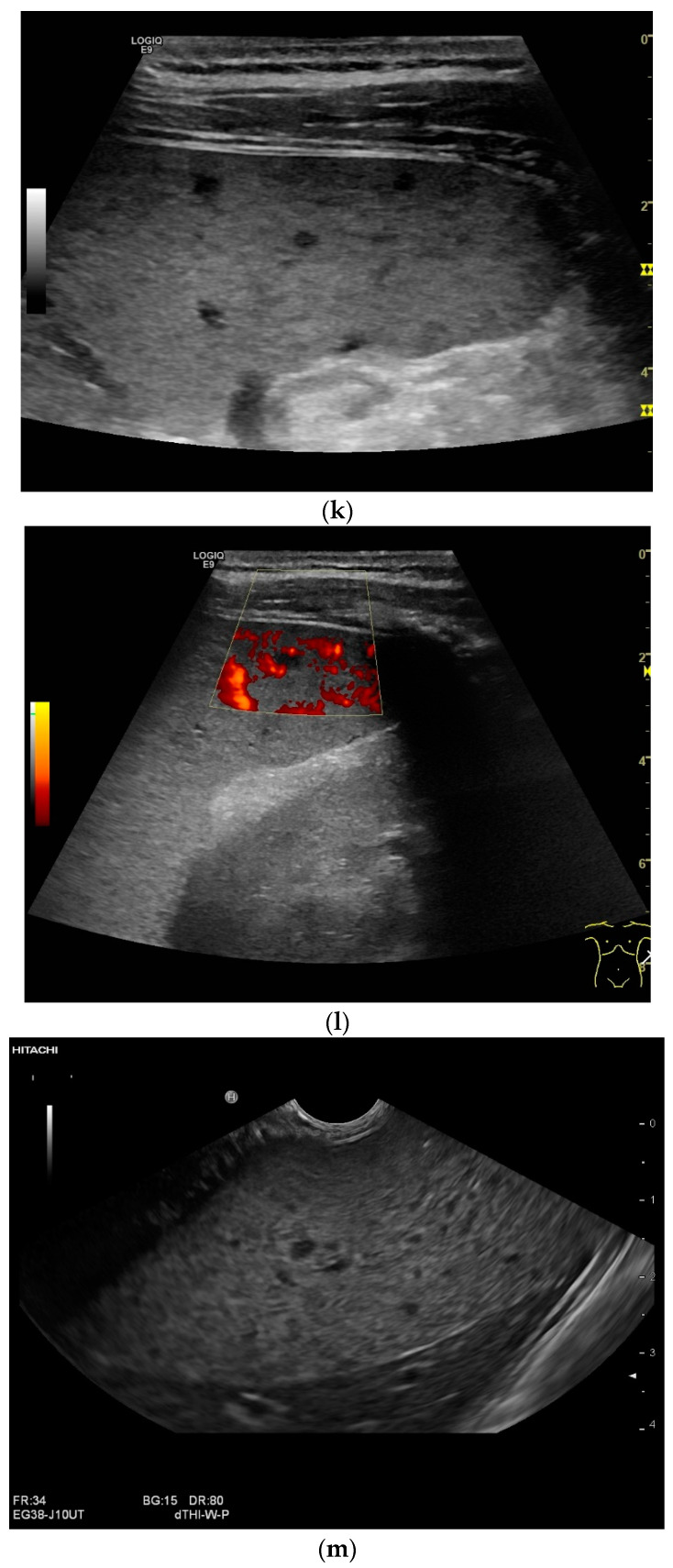
A case of extrapulmonary tuberculosis: 33 y/o male from a country at high risk of tuberculosis. Thoracic pain and fever led to a suspicion of pericarditis. There was no pericardial effusion. There were no pulmonary infiltrations or pleural effusions in the chest CT, but there were enlarged mediastinal lymph nodes. Ultrasound and CT also showed subdiaphragmal enlarged lymph nodes and small nodular splenic changes. The diagnosis was confirmed using the transgastric EUS-guided biopsy (19 G Olympus) of the lymph nodes in the hepatic hilum. Enlarged lymph nodes in the hepatic hilum (**a**); adjacent to the pancreatic head (**b**); transabdominal ultrasound (TUS) using a linear transducer of 9 MHz. The hypoechoic central parts are conspicuous (**a**,**b**). Rounded lymph nodes are observed as being peripancreatic (**c**) and as being in close proximity to the gallbladder wall (**d**). The lymph nodes are visualized between the markers. In the EUS, paragastric lymph nodes are enlarged, rounded, very hypoechoic, with hypoechoic central parts, and forming conglomerates. A central vascular hilum cannot be delineated in the CDI (**e**). The hypoechoic central parts are softer during elastography (**f**). EUS also shows enlarged hypoechoic lymph nodes in the hepatic hilus, with hypoechoic central parts that lack a central vascular hilum (**g**). Elastographically, the lymph nodes are indifferent (**h**). The diagnosis is confirmed using EUS-guided biopsy (**i**) with evidence of granulomatous inflammation, caseous necrosis, detection of acid-fast rods, and Mycobacterium tuberculosis in the PCR. In the spleen, single hypoechoic lesions < 5 mm are visible during transabdominal ultrasound using a linear transducer of 9 MHz. Otherwise, fine-grained hypoechoic lesions can only be guessed at (**j**). With magnification using a 9 MHz transducer, multiple hypoechoic lesions < 3 mm can be delineated (**k**). These do not reveal any vessels in the Power Doppler (**l**). EUS confirms multiple hypoechoic splenic lesions, in line with splenic tuberculosis (**m**).

Mediastinal lymph nodes can be biopsied using EUS- or EBUS-guided FNA or FNB. Via paraoesophageal, paragastric, and paraduodenal access, histological specimens can be obtained from further lymph nodes using EUS-guided sampling [41,43,45,46,47,48,49,50] [Figure 2a–m]. The most important differential diagnoses are metastases of malignant tumors, non-Hodgkin’s and Hodgkin’s disease and other infections. 

## 6. Liver

**Figure 3:**
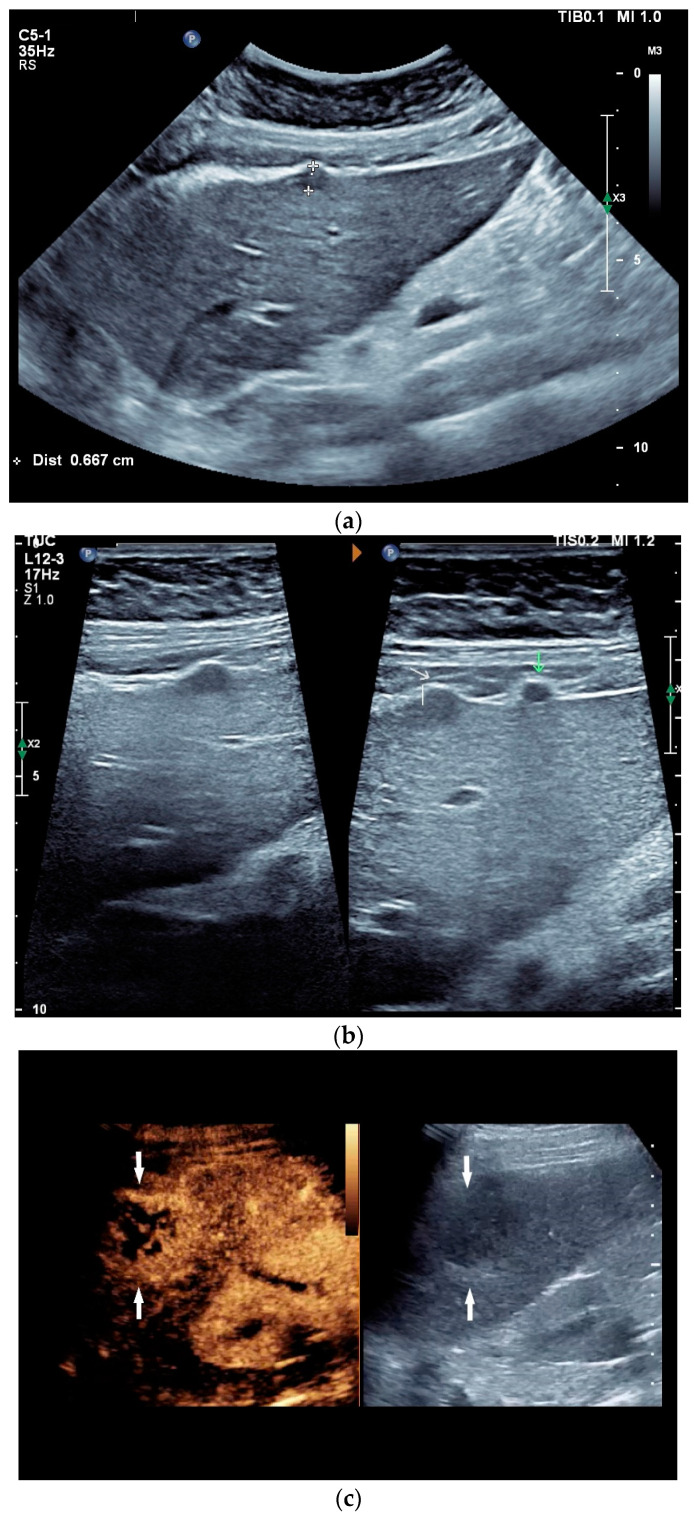

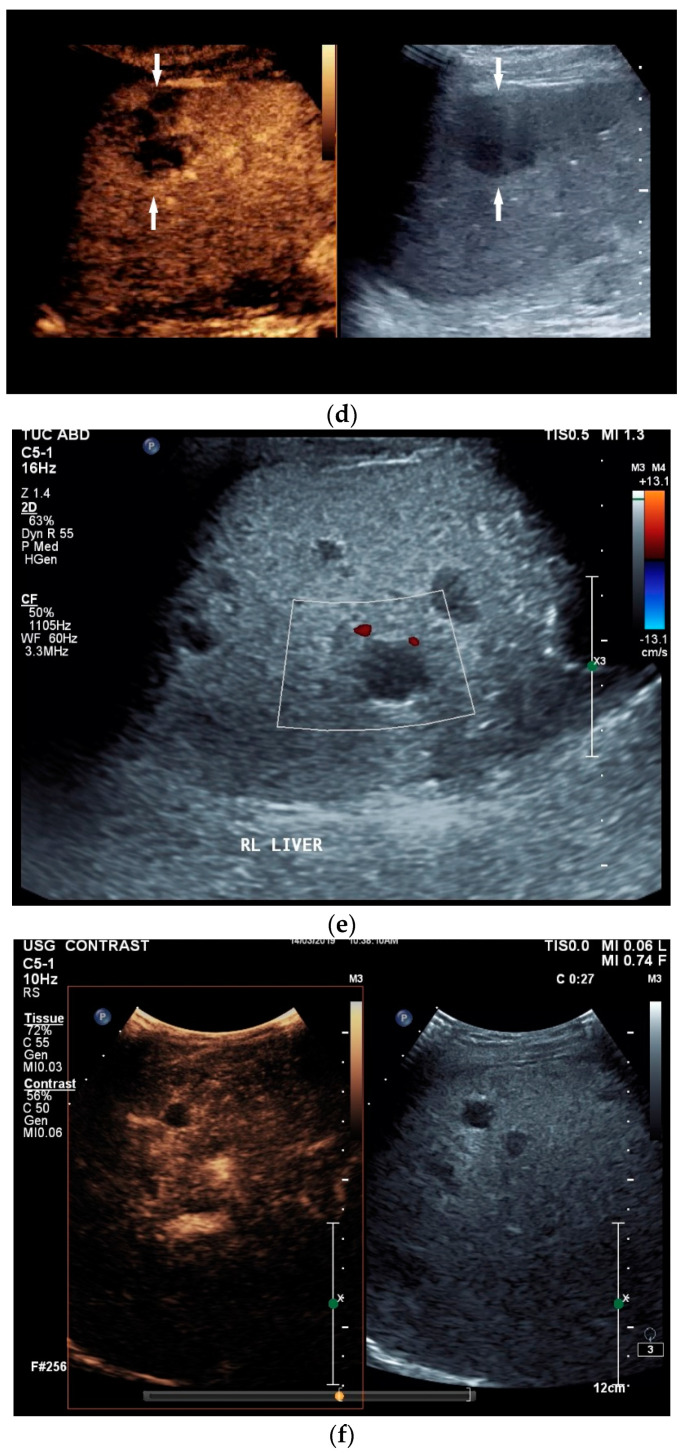

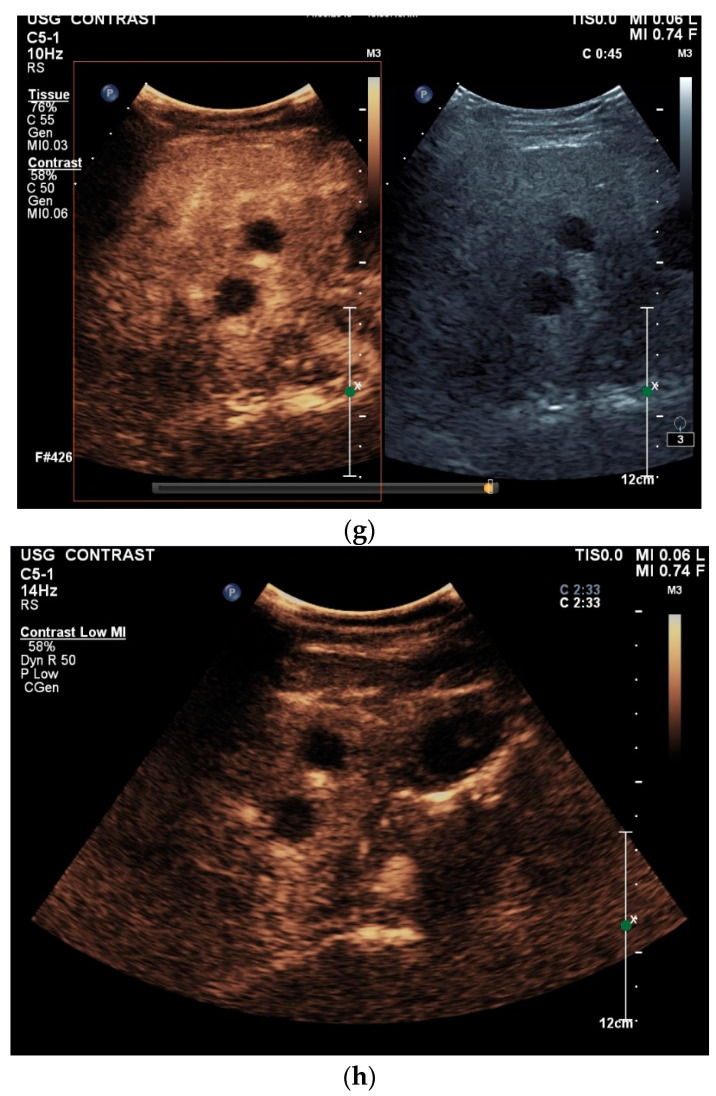
Sonographic and CEUS features of hepatic tuberculosis. Subcapsular liver lesion (**a**); subcapsular liver lesions (white arrow) and hypoechoic lesion on the capsule (green arrow) using a linear transducer of 12 MHz (**b**). Caseous necrosis, histologically proven. Hypoechoic lesion with hyperechoic rim using B-mode ultrasound (right side of image). The lesion is hyperenhanced in the arterial phase at the edge of the CEUS; the center is nonenhanced with the exception of some enhanced septa (**c**). In the portal venous phase, the peripheral areas are hypoenhanced (**d**). Multiple small, smoothly bordered, non-cystic hypoechoic lesions in the liver, with no evidence of macrovessels when using Color Doppler Imaging (**e**). CEUS shows mild peripheral enhancement around the lesions. The lesions are without enhancement in the arterial phase (**f**), portal venous phase (**g**), and late phase (**h**).

Liver involvement in active tuberculosis is rarely reported, with a frequency of approximately 1% [51,52]. It is more common with concomitant HIV disease at 18% [5,6,52]. Isolated tuberculosis of the liver is very rare, and has been described in just 1% of all cases [53,54]. In an autopsy study of patients with miliary pulmonary tuberculosis, 91% had liver involvement [55]. In the liver, tuberculosis manifests with diffuse- or focal-type nodular lesions. These can be micronodular (miliary) lesions or macronodular lesions [56,57,58]. A third variant is the serohepatic form, with a thickened liver capsule and subcapsular nodular lesions [56] [Figure 3a,b].

The micronodular form is usually caused by hematologic seeding via the hepatic artery in disseminated disease. If the nodules are very small and cannot be delineated via ultrasound, hepatomegaly sometimes remains the only conspicuous finding from liver ultrasound, with possible manifestations in other organs.

The macronodular form is characterized by single tuberculomas or macronodular lesions. Seeding more likely often occurs from the gastrointestinal tract via the portal vein [54]. A focal or nodular form without extrahepatic tuberculosis manifestation is very rare [59].

Abscesses can develop from the caseous necrosis, but are rare [58]. The formation of a multiseptated liver abscess with numerous septa has also been described in a case study [60] [Figure 3c–g].

The morphological correlate of hepatic tuberculosis is granulomatous inflammation, characterized by central caseous necrosis with peripheral granulation tissue. Both melting abscesses and calcified nodules may occur in the course of the disease.

Ultrasound presents hypoechoic or isoechoic liver lesions [56]. Hyperechoic lesions are rarely described (although, these can be found in hepatitis B infections and hepatitis C-related liver cirrhosis; however, histologically confirmed cases have categorized these as tuberculoma related, and not as regenerative nodule or hepatocellular carcinoma (HCC)) [59,61]. Depending on the stage of the disease, the inflammatory lesions are homogeneously hyperenhanced via CEUS. Most lesions develop a washout in the portal venous phase. Melting abscesses show a hyperenhanced rim with either a hypoenhanced or nonenhanced center or a heterogeneous enhancement [62,63] [Figure 3c,d]. Vascular complications, such as portal vein thrombosis, have been caustically described and found to be in connection with hepatitis B [54,64].

Important differential diagnoses are those of metastases, other granulomatous diseases with liver involvement, or other abscesses (mycotic, brucellosis). In the case of pre-existing liver disease, regenerative nodules and HCC must be included [6].

## 7. Bile Ducts

Biliary manifestation of tuberculosis is very rare. If large bile ducts are affected, wall thickening, and bile duct dilatation may be present. Intrahepatic strictures and bile duct dilatations indicate involvement of the intrahepatic bile ducts [6,65,66,67,68]. Diffuse miliary calcification along the course of the bile ducts has been described as a typical feature of tubercular cholangitis [56].

The infection of the biliary tract occurs via spreading from the portal system, from periportal lymph nodes, or ascending from the major papilla. Hematogenous seeding has also been described.

The most important differential diagnoses are primary sclerosing cholangitis, secondary sclerosing cholangitis, and cholangiocarcinoma [6,56].

## 8. Gall Bladder

Manifestation on the gallbladder may present as a nodule, mass, or circumferential thickening with homogeneous or heterogeneous enhancement [69]. Calcification may be indicative of tuberculosis, as well as the involvement of the neighboring liver or lymph nodes [70,71]. An irregular heterogeneously enhancing thickened gall bladder wall has been described as a manifestation of tuberculosis [72,73]. The most common differential diagnoses are gallbladder carcinoma or xanthogranulomatous cholecystitis [72].

## 9. Spleen

**Figure 4:**
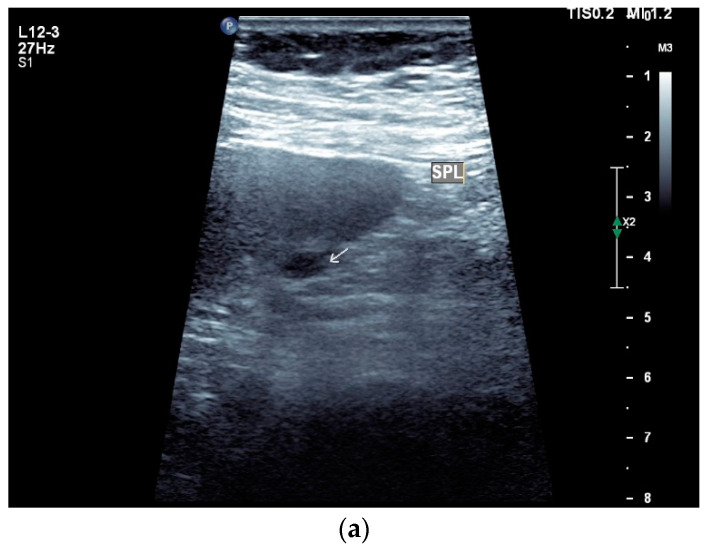

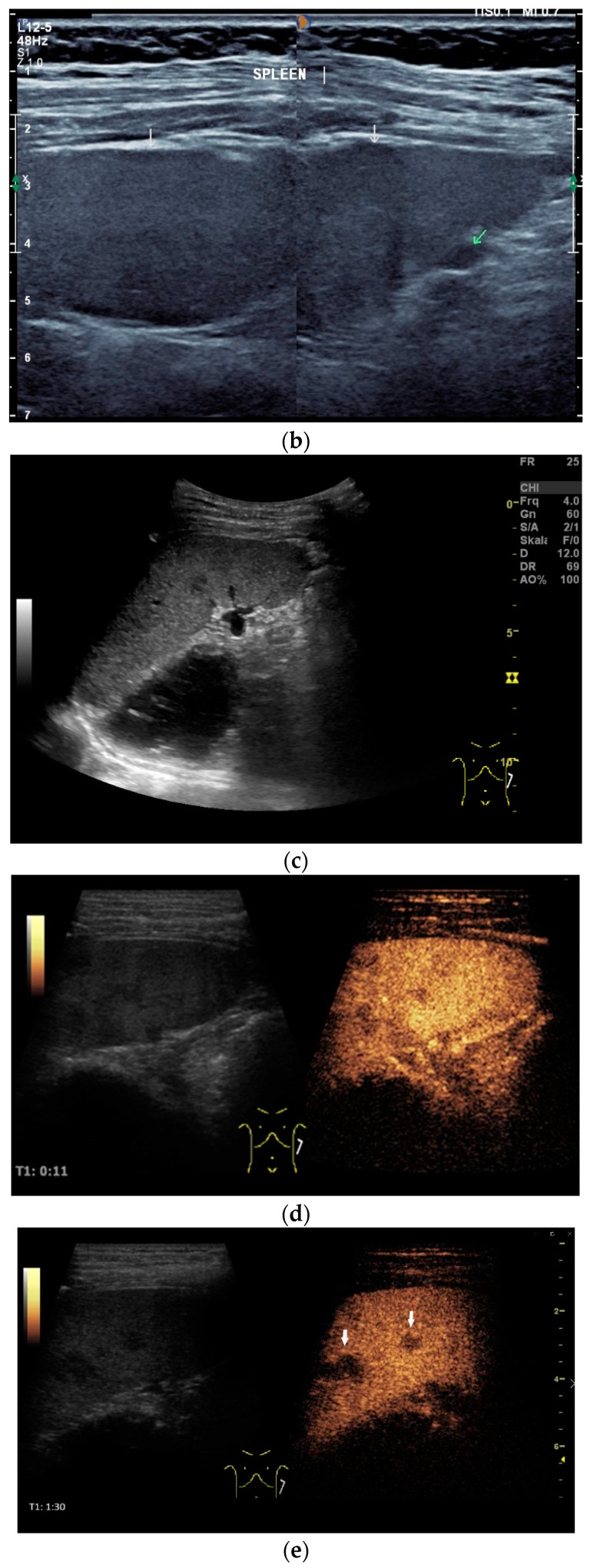

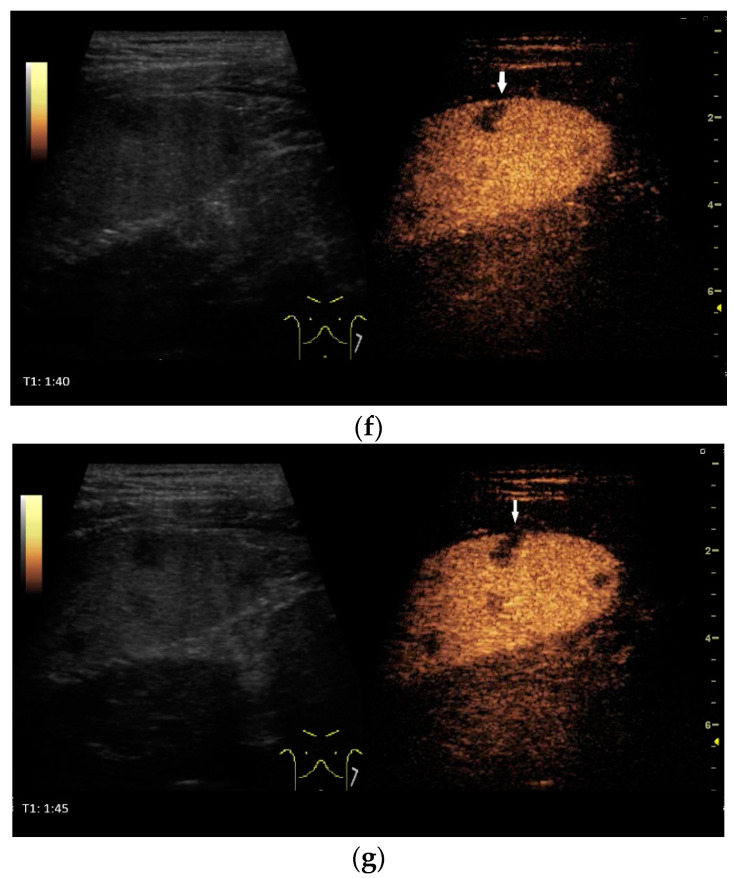
Sonographic and CEUS features of splenic tuberculosis. Sonographic and CEUS features of splenic tuberculosis. Subcapsular splenic lesion (arrow) (**a**), subcapsular splenic lesion using a linear transducer of 12 MHz (arrows) (**b**). Histologically confirmed tuberculosis from mediastinal lymph nodes. Multiple small splenic lesions (**c**). Using CEUS with a linear transducer (9 MHz), these are slightly hypoenhanced in the arterial phase (**d**), and show a progressive washout in the course of the venous phase (arrows) (**e**). Subcapsular splenic lesion (arrow) with nonenhanced and hypoenhanced parts and hyperenhanced rims indicate a caseous necrosis (**f**). In another section, an interrupted spleen capsule (arrow) due to a rupture of the caseous necrosis can be assumed (**g**).

Spleen involvement usually only occurs in disseminated disease. The manifestations are splenomegaly, hypoechoic micronodular (miliary), or macronodular lesions [8,74], as well as splenic abscesses [75] [Figure 4a–d]. In a group of patients with tuberculosis and mostly advanced HIV infection, 37% had splenic lesions [5]. In 33%, splenic lesions were associated with lymphadenopathy, and 16% each had splenic lesions and ascites or splenic lesions, ascites, and lymphadenopathy. Micronodular lesions may be indistinguishable. If this is suspected, examination with a high-resolution linear transducer is recommended. Using CEUS, splenic lesions in the arterial phase showed a hyperenhancement, followed by either a slow washout or a persistent enhancement in the middle and late parenchymal phase [76]. A fast washout was the exception, but nevertheless was also possible. These exceptional cases would therefore be difficult to differentiate from malignancies. There was also rim-like enhancement with central nonenhancement, like abscesses and septation-like enhancement [76]. Another study using CEUS in splenic manifestations of tuberculosis presented homogeneous enhancement (6.7%), heterogeneous enhancement (63.3%), and nonenhancement (30.0%). Most of the cases showed low enhancement. Among these cases, heterogeneous enhancement was divided into septal enhancement type (21.1%) and marginal enhancement type (78.9%) [77] [Figure 4c–g]. The most important differential diagnoses are non-Hodgkin’s lymphoma and other granulomatous inflammations, such as sarcoidosis, especially in the presence of lymphadenopathy. The splenic lesions must be differentiated from mycotic abscesses, leukemic infiltrates, and metastases. In the meta-analysis conducted by van Hoving et al. [78], splenic lesions as manifestations of abdominal tuberculosis had a broad sensitivity of 13–62% in five studies using B-mode ultrasonography. The specificity was higher, at 86–100%.

## 10. Pancreas

**Figure 5:**
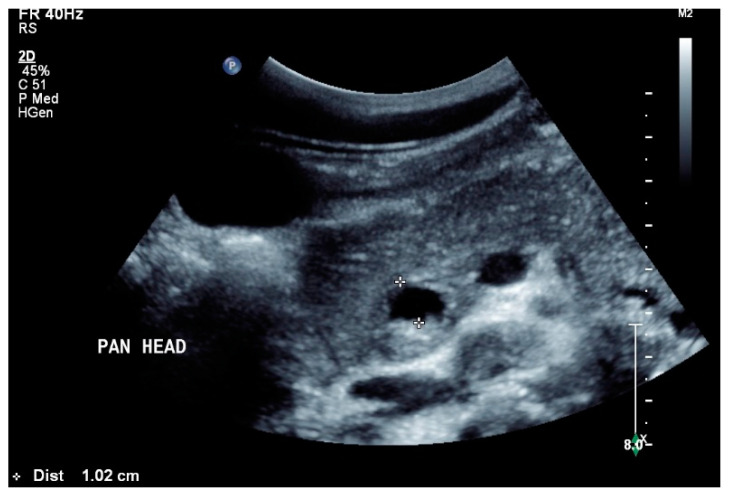
Pancreatic tuberculosis. Plump large pancreas. There is a lesion with a thick echogenic capsule and hyperechogenic and anechoic contents (between the markers) at the head of the pancreas. This finding was evaluated as a caseous abscess in a patient with tuberculosis.

In a retrospective analysis of 384 patients with abdominal tuberculosis, 8.3% had pancreatic manifestations. In more than 50% (18/32), the pancreas with peripancreatic involvement was the primary manifestation of tuberculosis. Furthermore, 53% were HIV-positive, but only 15% presented amylase or lipase elevation [79]. Ultrasound showed a diffusely heterogeneous pancreas in 15%, as well as solitary or multiple pancreatic lesions in all patients [Figure 5]. Most lesions were hypoechoic (90.6%), and few were isoechoic (9.4%). Multiple lesions were detected in 37.5%, and solitary lesions in 63.5%, respectively. The pancreatic duct was not generally dilated [79]. Additionally, 75% of patients had concomitant peripancreatic lymphadenopathy. A characteristic feature of these lymph nodes were the hypoechoic central parts [79]. EUS showed hypoechoic or mixed cystic solid lesions, as well as often showing calcifications. Using EUS, peripancreatic lymph nodes as well as mediastinal lymph node enlargements were detected simultaneously [80,81,82,83,84]. Both the pancreatic lesions and the peripancreatic and mediastinal lymph nodes can be subjected to EUS-guided biopsy [46,83,85]. In a study of 12 patients with solitary pancreatic tuberculosis manifestations, typical imaging findings when using CEUS were hyper- or isoenhancement in the arterial phase, and hypoenhancement in the late phase [83]. Using CH-EUS, the examined lesions showed hyperenhancement. In ultrasound elastography and EUS elastography, the tuberculosis lesions were stiffer than the surrounding area. EUS elastography showed that the stiffness was dependent on the stage of the disease [83]. The common bile duct was not dilated [83]. Using EUS-FNA, pancreatic/peripancreatic tuberculosis was correctly diagnosed in 76.2% of cases [86]. Diagnostically significant in the EUS-FNP are granulomatous inflammation, a positive tuberculosis polymerase chain reaction test, Ziehl–Neelsen staining, and positive cultures of Mycobacterium tuberculosis [86]. A calcified lesion with a cheesy core observed during macroscopic on-site evaluation should be examined for tuberculosis [46]. The most important differential diagnoses are pancreatic ductal adenocarcinoma, pancreatic neuroendocrine neoplasia (PanNEN), metastases, autoimmune pancreatitis and lymphoma manifestations [83,85,87], or cystic neoplasms [88].

## 11. Gastrointestinal Tract

The entire gastrointestinal (GI) tract from the esophagus to the rectum can be affected by tuberculosis, and about 11% of patients with extrapulmonary tuberculosis present involvement of the GI tract [89].

**Ileocecal region:** The most frequent localization remains the ileocecal area (76–90%) [3,36,90]. The predilection in the ileocecal region is attributed to the high density of lymph follicles in this area [36]. In a study conducted by Barreiros et al., 100% of patients with intestinal tuberculosis presented intestinal wall thickening. Intestinal tuberculosis was localized predominantly on the right side in the terminal ileum and cecum, and less frequently also in the ascending colon. Enlarged mesenteric lymph nodes were found in 87%, as well as in 43% of fistulas [91]. Macroscopic, ulcerating, hypertrophic, or ulcerating-hypertrophic (mass forming) changes are described in the gastrointestinal tract. In the ileocecal area, wall thickening is observed, which can be symmetrical or asymmetrical. The regional lymph nodes may be enlarged [3]. The wall is markedly thickened in most cases between 1–2 cm, and is hypoechoic in all patients [36]. An instance of the pseudo-kidney sign was also observed in one patient. This corresponded to tuberculosis, rather than a suspected tumor [36].

In CEUS, two types of enhancement have been observed. Type 1 (13%) showed enhancement first in the serosa, then in the mucosa. Type 2 (87%) exhibited simultaneous enhancement of the entire wall. Histopathologically, type 1 presented lower levels bowel wall damage and a fewer number of granulomas when compared to type 2. In type 2, most patients had heterogeneous enhancement, and few had homogeneous enhancement. Heterogeneous enhancement correlated with caseous necrosis [36]. The arrival time for the contrast medium in the tuberculous intestine was 14.8 ± 1.3 s, and was therefore faster than in the healthy intestine [36]. All patients with tuberculosis in the ileocecal region had enlarged lymph nodes with destroyed lymph node hilum and hypo- to anechoic central lymph node areas, corresponding to caseous necrosis [36]. While fistulas and strictures were not reported in the study by Yang et al. [36] they were present in a few patients in the study conducted by Ma et al. [92].

Gastrointestinal ultrasound can be used to monitor treatment response [92]. The most important differential diagnoses are Crohn’s disease, lymphoma manifestations, and carcinomas.

**Figure 6:**
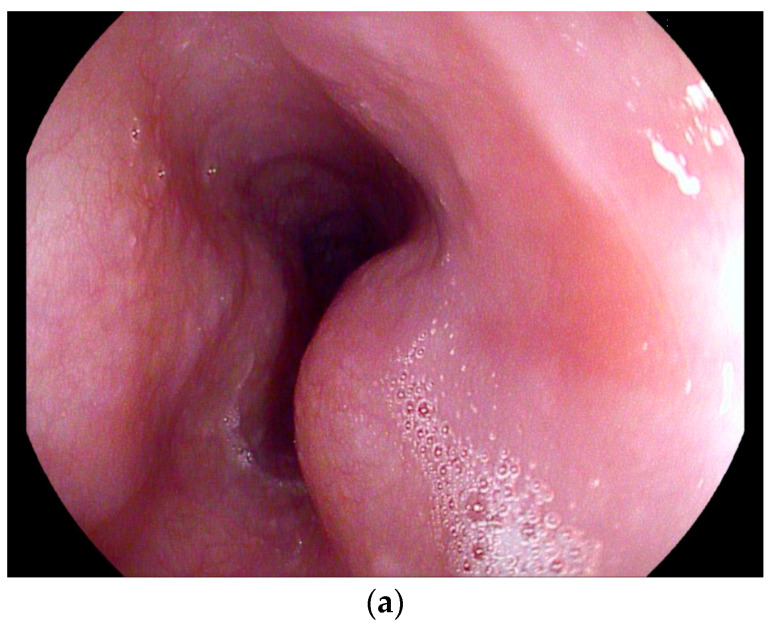

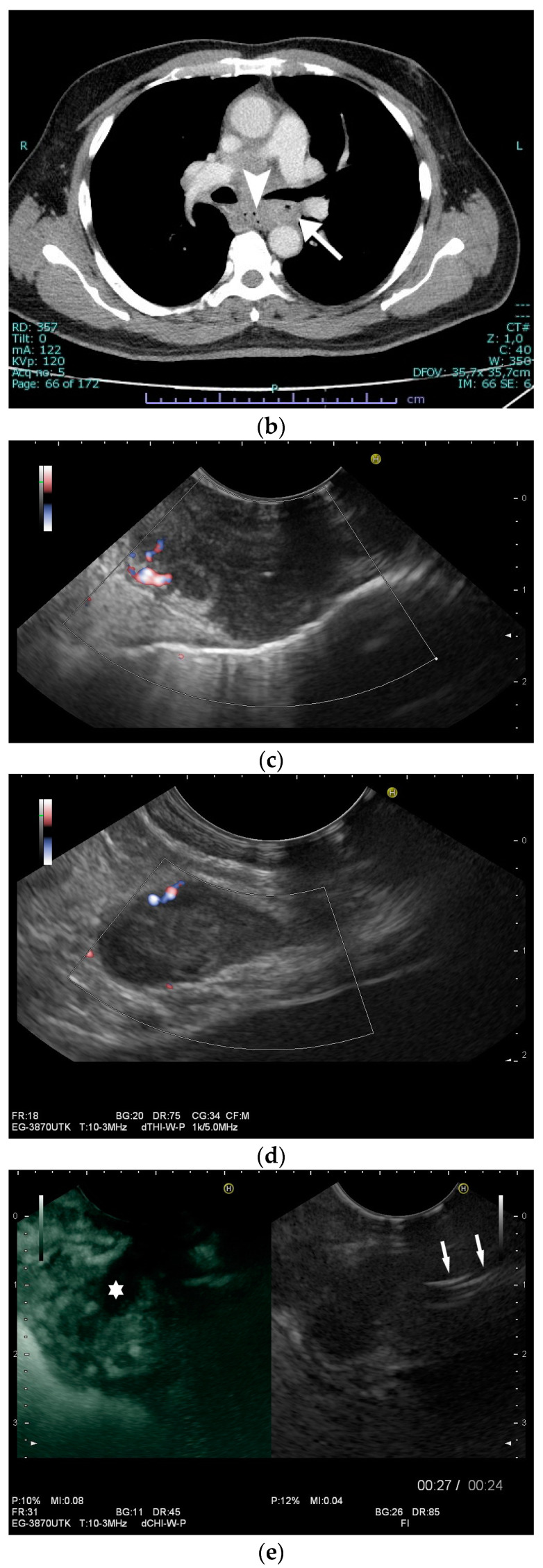

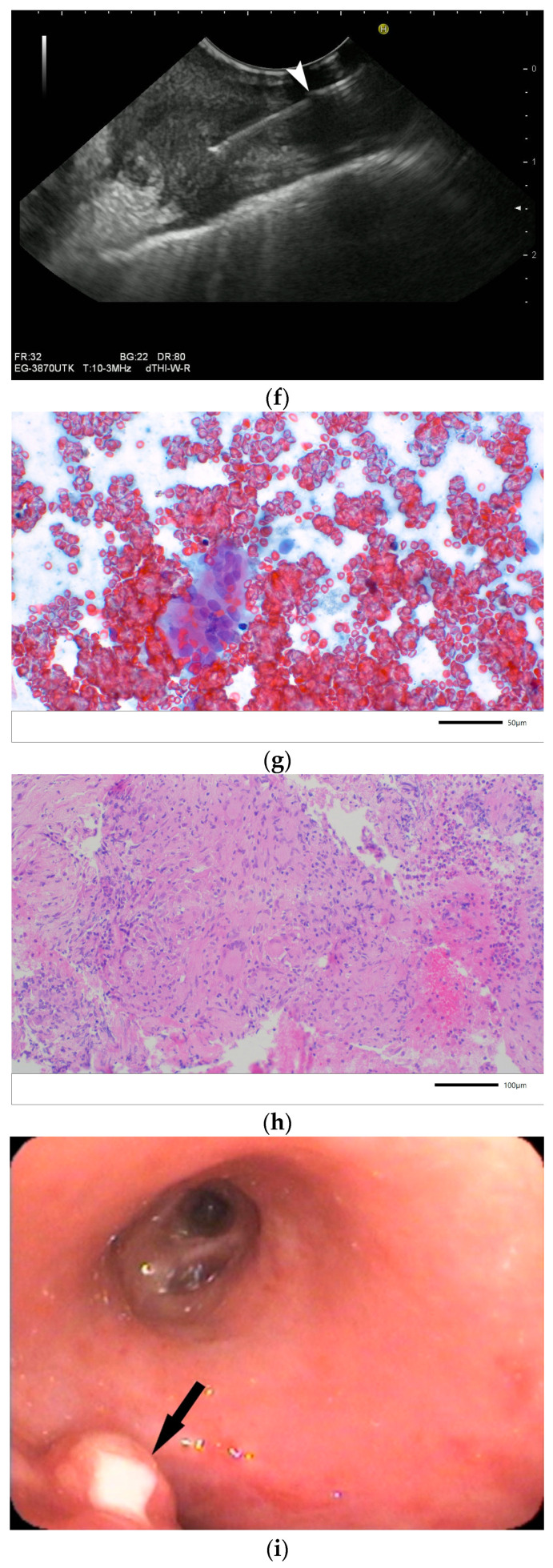
A case of esophageal tuberculosis. A 52-year-old male non-smoker presented with progressive dysphagia and weight loss (15 kg in 6 months). Upper GI endoscopy revealed two subepithelial esophageal masses covered by normal mucosa (**a**). A CT scan showed large solid mass lesions in the mediastinum, with small gaseous inclusions (arrowhead) and a thickened esophageal wall (**b**, arrow). EUS ruled out a subepithelial esophageal tumor, and confirmed a large mediastinal hypoechoic mass infiltrating the esophageal wall, which was up to 12 mm thick with a complete loss of layering (**c**). Multiple enlarged and confluent hypoechoic lymph nodes were found throughout the mediastinum (**d**). Contrast-enhanced harmonic EUS showed the hyperenhancement of the mediastinal mass lesion and the thickened esophageal wall with some anechoic necrotic parts (*), and echogenic gaseous reflections (arrow; **e**). EUS-FNA (22 Gauge) of lymph nodes and of the thickened wall was performed (**f**; needle is marked with an arrowhead). Turbid fluid was aspirated from one lymph node, and then sent for cytological and microbiological examination and polymerase chain reaction (PCR) for mycobacteria. Positive Ziehl–Neelsen staining and PCR for mycobacterium tuberculosis and cheesy, necrotizing granulomas (**g**), cytology, Papanicolaou stain: giant cell; (**h**), histology, hematoxylin-eosin stain: necrotizing granulomas; courtesy Gunnar Schröder, Institute for Pathology Wildau, Germany) established the diagnosis of extrapulmonary tuberculosis involving the mediastinum and esophagus. Bronchoscopy revealed a fistula opening in the left main bronchus marked by the black arrow (**i**).

**Esophagus:** Esophageal tuberculosis accounts for <0.2% of all tuberculosis patients, and for only 2.8% of all gastrointestinal cases [89,93]. In 2022, two systematic reviews analyzed 133 and 311 cases of esophageal tuberculosis [94,95]. Esophageal tuberculosis is secondary to tuberculous mediastinal lymphadenopathy, which causes infiltration, ulcerations, bulging lesions, and narrowing of the esophageal wall [45,96]. Due to direct extending and spreading from infracarinal nodes, the middle third of the esophagus is usually affected [97,98]. Patients most often present dysphagia and retrosternal pain. Due to these symptoms and the accompanying weight loss, esophageal cancer is frequently suspected [99]. Complications of esophageal tuberculosis include bleeding, perforation, aspiration pneumonia, hematemesis, traction diverticula, esophageal strictures, and fistula formation [100]. In about 50% of cases, characteristic features of pulmonary involvement are detected during radiological imaging, and may help to establish the diagnosis [101]. Otherwise, the symptoms prompt esophagogastroduodenoscopy, with biopsy as the first diagnostic modality. Remarkably, esophageal tuberculosis can lead to variable endoscopic findings, ranging from esophageal ulcers or multiple nodules to a hypertrophic growth like an esophageal polyp, a subepithelial tumor, or tumor-like lesions [102]. Differential diagnosis should include sarcoidosis and Crohn’s (if an epithelioid granuloma without caseous necrosis is present), or carcinoma, lymphoma, and Behcet’s disease must be considered. Since caseating granulomas are usually located deep in the submucosal layer of the esophagus, endoscopic mucosal biopsies have a sensitivity of only 22%, with classical granulomas only being detected in half of the patients, and acid-fast bacilli only in one of four patients [102]. Endoscopic ultrasound most often visualizes infracarinal lymph node conglomerates with hypoechoic central lymph node areas [103] and hypoechoic homogeneous or heterogeneous lesions of the esophageal wall, with the possible interruption of the adventitia [45,96,104]. Fistula formation is possible [105]. Endoscopic ultrasound (EUS) and EUS-guided sampling play a pivotal role in confirming the diagnosis [103] [Figure 6a–i].

**Stomach:** The prevalence of gastric tuberculosis is reported as 0.5%. It usually results from the dissemination of pulmonary tuberculosis [45]. Hypoechoic malignant suspected ulcerations or protruding lesions in the wall [106] are often caustically described, as well as abscesses in the submucosa [107].

**Duodenum:** The prevalence in the duodenum is observed to be around 2.0–2.5% of all manifestations in the gastrointestinal tract [101]. Complications include strictures and fistula formation [108].

**Jejunum**: A manifestation in the jejunum can occur with single or multiple short or long segmental strictures. Isolated involvement of the jejunum is rare [101]. The perforation of the small intestine may occur as a further complication [109].

## 12. Pericardium

The detection of a pericardial effusion using ultrasound in patients with tuberculosis is associated with an increased risk of pericardial tuberculosis. Therefore, the assessment of a pericardial effusion should always be included in the ultrasound tuberculosis workup [5]. Up to 2% of people suffering from pulmonary tuberculosis have tuberculous pericarditis [110]. In Africa, pericardial diseases are most commonly caused by tuberculosis [111]. The development can lead to pericardial tamponade [111,112]. In a study from an endemic area, 53% of patients with pericardial effusions of 10 mm or more had tuberculous pericarditis; 45.5% were HIV-positive [113]. In the chronic form of pericardial effusion, fibrin and septal cords may be present. Tuberculous pericarditis can lead to constrictive pericarditis, which can be characterized during echocardiography by pericardial thickening, calcifications, septal bounds, and the impaired diastolic filling of the heart [8,110].

## 13. Urogenital System

### 13.1. Kidneys, Ureter, Urinary Bladder, and Urethra

The urogenital system is affected in 15–20% of extrapulmonary infections [114]. About 4–8% of patients with pulmonary tuberculosis develop a clinically significant genitourinary infection [114]. Renal tuberculosis is the result of the hematogenous seeding of M. tuberculosis into the glomerular and peritubular capillary bed from a pulmonary site of primary infection. The latency period between the initial infection and renal manifestation can be several years. Granulomas arise next to the glomeruli. In the course of the disease, caseous necrosis, caverns, calcifications, fibrosis, and strictures occur. Papillary necrosis and medullary cavities, fibrosis with stenosis, and dilatation of the calices can also develop. Infundibular strictures can lead to localized caliectasias [114]. Parenchymal scars are common, and the parenchyma may be narrowed, either locally or generally. Extensive calcifications may be present [114], and can be arranged in a diffuse miliary or triangular lobar pattern [115]. The lobar pattern of calcification is pathognomonic for tuberculosis [114,115]. Fine calcifications can also be found in the walls of the calyces, pyelon, or ureter [115]. However, urethral calcifications can be seen when using ultrasound below the pyelon, or before they enter the bladder. In CEUS, the thickening of the visible ureter in tuberculosis proved to be both homogeneous and heterogeneous, and predominantly hypoenhanced [116].

**Figure 7:**
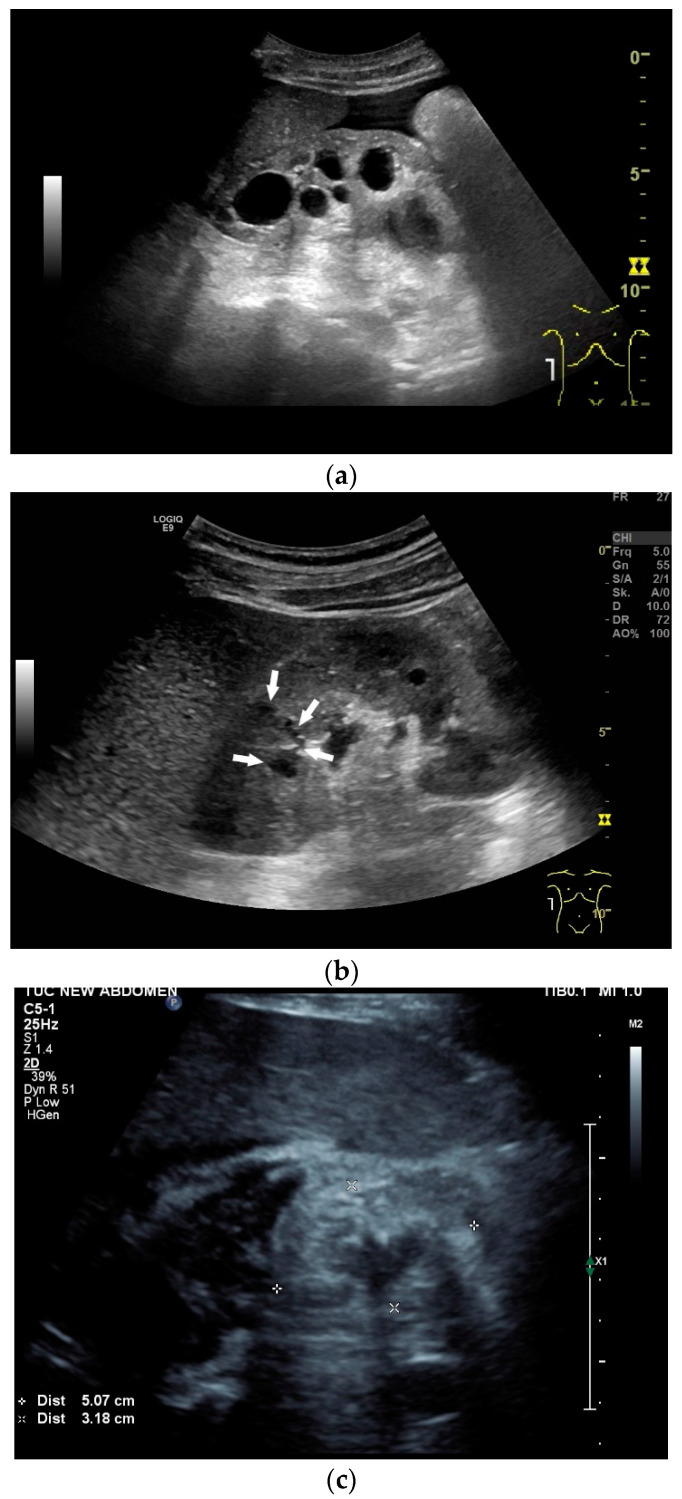

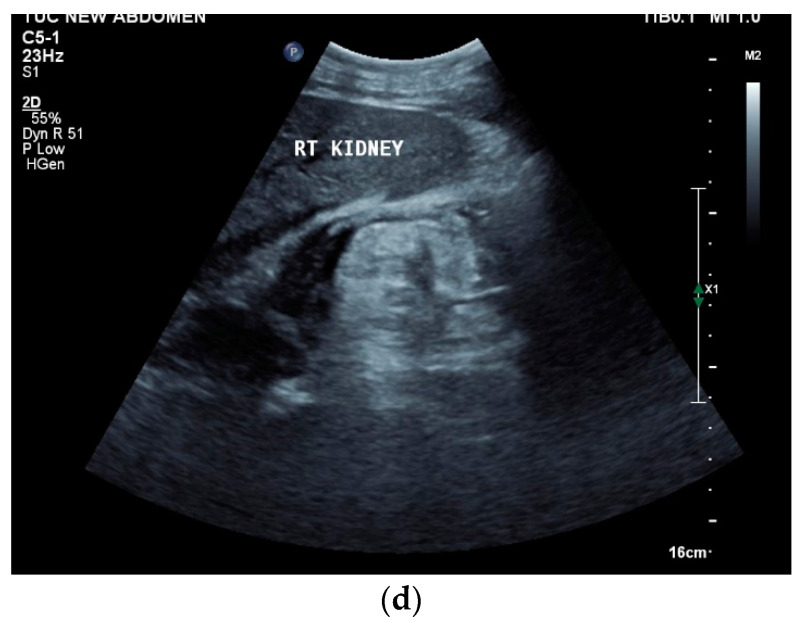
Renal tuberculosis. Adjacent to the renal sinus are cyst-like lesions with smooth borders. Characteristic of caliectasias (**a**). Small “tubular” structures are noticeable in the kidney (arrow). The parenchyma presents nodular hypoechoic lesions. Perforation of caseous necrosis, and small intrarenal fistulas (**b**). “Putty kidney”—final stage of renal tuberculosis. Very small (non-functioning) kidney with highly diffused hyperechoic parenchyma (**c**), surrounded by a perinephritis fluid collection (**d**).

Papillary necrosis can occur, caliectasias develop in the course of the disease, and some patients develop hydronephrosis [114]. Caliectasia can present as asymmetric hydrocalicosis [115] [Figure 7a,b].

“Putty kidney” is a possible final stage of renal tuberculosis. The term describes diffuse parenchymal renal calcifications that can be seen on a simple X-ray, as well as a “putty-like” substance, which is found inside a surgically removed tuberculosis kidney [117,118] [Figure 7c,d].

If the ureter is affected, mucosal irregularities and ureteral dilatation due to localized or multilocular strictures must be differentiated in radiological imaging. The ureter can shorten (pipe-stem ureter) [114]. Varied effects of tuberculosis on the urinary tract are infundibular stenosis with caliectasis, moth eaten calyx, parenchymal calcification, cortical scarring, caseous mass/abscesses (with potential rupture into the perinephritic space and calyx) [Figure 7b], a hiked-up pelvis, urothelial thickening, and a beaded ureter [115].

The urinary bladder may be reduced in size (shrunken), and may only have a small volume. The wall is thickened, and may be irregular due to the granulation tissue. Like the ureter, the urethra may also have strictures. In advanced disease, the bladder is referred to as a “thimble bladder” [115].

Focal renal parenchymal changes in inflammatory renal diseases can be difficult to detect using ultrasound [115,119]. However, carefully performed sonographic examinations with knowledge of the possible pathology can visualize most changes. In a large series, the diagnosis using ultrasound was confirmed in 59% of cases [120].

A common parenchymal change is the granuloma. Small (5–15 mm) focal lesions are either hyperechoic, or have an echogenic rim with a central area of low echogenicity. Larger focal lesions (>15 mm) are heterogeneous and have poorly defined margins. Color Doppler Imaging methods allowed vessel ruptures at the lesions to be observed. Caseous necroses are hypoechoic or anechoic with hyperechoic margins. The rupture into the calices or perirenal space presents as a narrow hypoechoic road that can be detected via careful examination, especially with a high-resolution transducer. This requires a good knowledge of the changes to be expected. Fine septa can be present in the caseous necroses. Focal caliectasia, secondary to infundibular stenosis, is common, often being associated with varying degrees of urothelial thickening. This can result in a quite characteristic sonographic pattern of a focally dilated anechoic calyx with hyperechoic debris masses. If one pays close attention, the urothelial thickening in the calices can be well delineated [115]. To the best of our knowledge, there are no CEUS studies of granulomas and caseous necrosis in the kidneys. However, CEUS has been shown to increase the diagnostic accuracy of ultrasound imaging in infectious kidney diseases [119,121]. Similar to other focal melting inflammations, it can be assumed that the caseous necroses are nonenhancing when using CEUS. Other important differential diagnoses of renal tuberculosis manifestations are melting inflammations, tumors, atypical cysts, and xanthogranulomatous pyelonephritis.

### 13.2. Adnexa, Fallopian Tubes, Uterus

**Figure 8:**
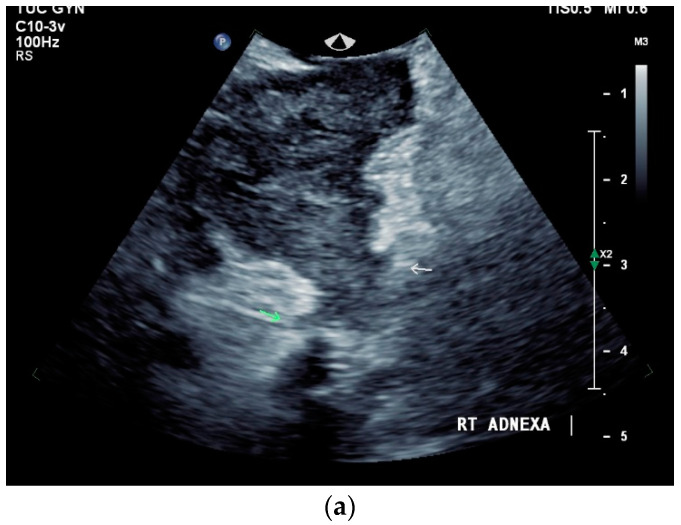

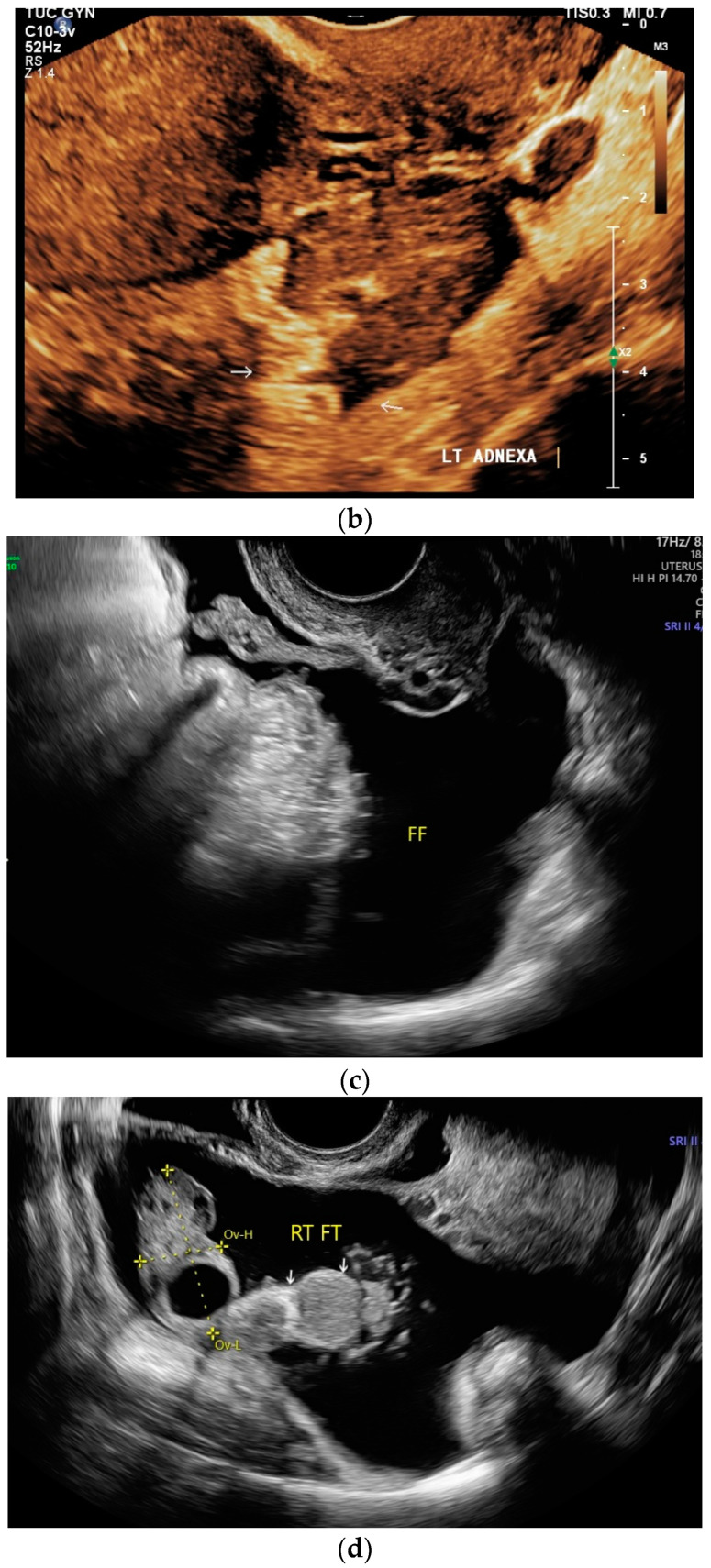

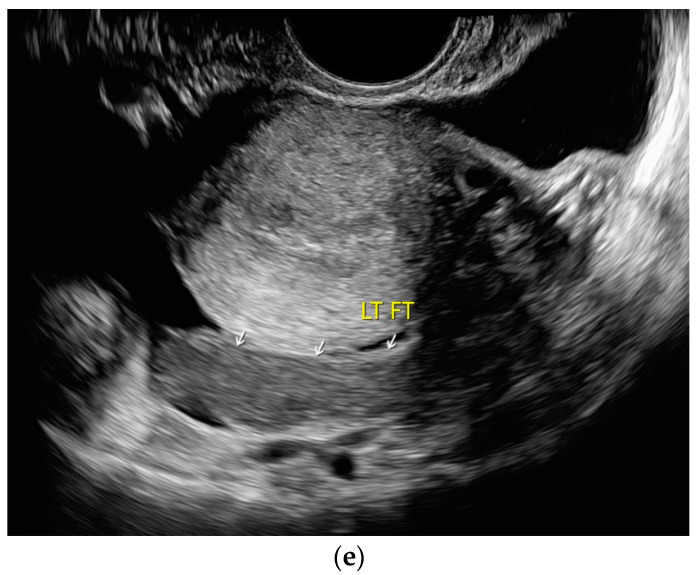
Tuberculosis of the uterus and adnexae. Female patients with tuberculosis in India. Pelvis with tubo-ovarian masses: Hypoechoic lesion in right adnexa (arrows) (**a**) and hypoechoic lesion in left adnexa and a small amount of free fluid (arrows) (**b**). Free fluid in the Douglas space (arrows) (**c**). Thickened right fallopian tube (RT FT) (arrows) (**d**); thickened left fallopian tube (LT FT) (arrows) (**e**).

Signs of tuberculosis in abdominal or vaginal ultrasounds in the female genital area are ovarian cyst, tubo-ovarian mass, hydrosalpinx, pyosalpinx, adhesion, adnexal fixation, thin endometrium, endometrial fluid, endometrial calcification, endometrial synechiae (Asherman’s syndrome), cornual synechiae, disturbed endometrial vascularity, ascites in the small pelvis, and peritoneal or omental thickening [122]. In a study with 175 infertile patients, ovarian cysts (23.4%) and tubo-ovarian masses (15.4%) were the most common manifestations [122] [Figure 8a–e].

### 13.3. Epididymis

In the case of tuberculosis manifestations of the epididymis, it is predominantly diffusely enlarged, and appears to hypoechoic when using ultrasound. Focal changes are less common. As a rule, the changes are unilateral [123].

### 13.4. Testes

Infections of the testes are usually associated with an infection of the epididymis. Ultrasound imaging presents diffuse or focal hypoechoic areas. These must be differentiated from lesions of other origins and tumors in particular. The combination with an enlarged hypoechoic epididymis makes a malignant genesis rather unlikely [123].

### 13.5. Prostate

Caseous necrosis and calcifications are frequent pathological phenomena of prostate tuberculosis. Caseous necrosis must be differentiated from abscesses of a different origin [123]. When using transrectal ultrasound (TRUS), prostatic enlargement, hypoechoic lesions, and calcifications were described in cases of tuberculosis manifestation in the prostate. However, these changes were non-specific, and also occurred in men without tuberculosis [124]. In contrast-enhanced TRUS (CE-TRUS), the tuberculosis lesions were found to be hypoenhanced and nonenhanced. More lesions were detected when using CE-TRUS than in the native TRUS. Caseous necroses were both hypoenhanced and nonenhanced, depending on the stage of development. The remaining hypoenhanced lesions corresponded to tuberculous granulomas and the incomplete destruction of the glands [124].

### 13.6. Vas Deferens

The vas deferens is a direct continuation of the epididymal tube. There are three different types of vas deferens tuberculosis: the uniformly thickened wall type, the tuberous type, and the abscess type. When passing through the inguinal canal, the tuberous type of vas deferens tuberculosis must be differentiated from inguinal lymph nodes of various origins [125]. During ultrasound, the lesions are predominantly 1–3 cm in size, but smaller and larger lesions are also observed. The lesions are mostly hypoechoic or heterogeneous. They form conglomerates. Less than a fifth have calcification [125]. Vas deferens tuberculosis lesions have proven to be heterogeneously and diffusely enhanced via CEUS [125]. Heterogeneous enhancement was categorized as septal, annular, or nodule-in-nodule enhancement [125].

### 13.7. Thyroid

The thyroid manifestation of tuberculosis is usually secondary to manifestations in other organ systems. The infection of the thyroid gland occurs from surrounding diseased lymph nodes or hematogenous spreading. Primary infection is very rare, accounting for less than 1% of all thyroid tuberculosis cases [126]. Thyroid tuberculosis can also be termed as tuberculous thyroiditis. There are three different types: the granulomatous type, the caseous type, and the diffuse type. The first two types in particular correspond to the stage of the disease [126]. The sonographic imaging is described casuistically. Patients with primary thyroid tuberculosis who underwent surgery for suspected malignancy had preoperative malignancy-like changes during ultrasound imaging. The retrospective analysis described lesions with indistinct borders and no obvious blood flow signal within the mass; or lesions surrounded by a strong echo or by a predominantly mixed echo; or lesions with a scattered strong echo in some parts. In addition, macrocalcifications, sickle-shaped, or ring-shaped calcifications were visible [126]. Other investigators describe the enlargement of the thyroid lobes and mixed cystic and solid hypoechoic lesions [127], anechoic lesions with irregular borders [128], or large, heterogeneous, mostly anechoic lesions with irregular vascular walls and a small amount of internal echo [129]. The appearance may change under therapy. The nodes shrink, become solid, and may show calcifications [129].

## 14. Diagnostic Accuracy of Ultrasound in the Diagnosis of Extrapulmonary Tuberculosis Manifestations

The aim of our overview was to describe and explain the typical but also very diverse appearance of extrapulmonary tuberculosis manifestations in order to facilitate the recognition and diagnosis of tuberculosis manifestations. However, the question arises as to how reliably a diagnosis of tuberculosis can be made on the basis of ultrasound findings. Or how reliably can extrapulmonary tuberculosis be ruled out if the ultrasound is unremarkable? The microbiological or molecular detection of Mycobacterium tuberculosis is considered to be the reference standard in the diagnosis of tuberculosis. However, the test is not always available or remains (false) negative [130]. In addition to clinical symptoms, however, conspicuous findings from ultrasound, X-ray, and computed tomography examinations are crucial in order to raise suspicion of unusual tuberculosis manifestations, and to enable the collection of adequate samples for microbiological and molecular diagnostics. However, ultrasound findings are not highly specific, and may have other causes. In a COCHRAN analysis comprising 11 studies and 1319 participants, both a higher-quality and a lower-quality reference standard were utilized to assess the accuracy of abdominal ultrasound imaging in the diagnosis of tuberculosis among HIV-positive people with suspected abdominal tuberculosis or disseminated tuberculosis with abdominal involvement [78]. For the studies with a higher diagnostic standard (microbiological confirmation), ultrasound had a pooled sensitivity and specificity of 63% (with a range of 35–82%) and 68% (with a range of 20–92%), respectively. In the studies with lower diagnostic quality standards (based on clinical appearance and imaging, but without microbiological confirmation), both pooled sensitivity and specificity were higher, at 68% (a range of 37–88%) and 73% (a range of 22–92%), which was attributable to the fact that the inclusion of ultrasound findings in the diagnostic criteria for tuberculosis represents a diagnostic bias. The heterogeneity of the diagnostic performance of ultrasound for the various organ manifestations may be explained by several factors. These include the different quality of equipment used in studies published from 1997 to 2019, the different examination protocols (Point-of-care ultrasound [PoCUS] using the FASH-protocol and low-end ultrasound systems, as well as comprehensive ultrasound in tertiary centers using high-end equipment), as well as the differences of tuberculosis prevalence in the included cohorts, ranging from 17.5% to 71%. Van Hoving et al. calculated the following scenario: in 1000 HIV patients with a 20% prevalence of tuberculosis, 74 of 200 tuberculosis cases are missed, and 256 patients are incorrectly diagnosed and over-treated as tuberculous, based on ultrasound criteria alone [78]. In turn, without ultrasound, a therapy that is necessary but not carried out would endanger those suffering from tuberculosis and their environment. In conclusion, ultrasound alone is neither sufficient to rule out or confirm abdominal tuberculosis. The authors of the meta-analysis recommend including sonography together with microbiological and laboratory diagnostics and X-rays in the diagnostic algorithms [78]. In a prospective study in India, presumptive tuberculosis patients with and without HIV underwent PoCUS in order to assess pericardial, pleural, and ascitic effusions, abdominal lymphadenopathy, and hepatic and splenic micro abscesses. In the HIV-negative patients, PoCUS results did not correlate with tuberculosis in one third of patients. They had either other infectious diseases or tumors [131]. For the diagnosis of tuberculosis in HIV patients in an emergency center in South Africa, it was shown that the FASH-combined protocol had sensitivities and specificities of 71% and 57%. The diagnostic sensitivity for individual ultrasound features was between 3 and 47%. The specificity, on the other hand, was between 75 and 99% [132].

## 15. Conclusions

Extrapulmonary manifestations of tuberculosis are not uncommon. The sonographic appearance reflects the stages of the disease with granulomatous inflammation, caseous necrosis and abscesses, septations, fibrosis, shrinkage, and calcifications. CEUS findings are closely related to the disease stage. Initially, heterogeneous hypervascularization appears in the stage of granuloma formation. Central nonenhancement with marginal rim enhancement is seen in the stage of the formation of caseous necrosis and abscesses. The visualization of typical ultrasound changes is essential for the detection of extrapulmonary manifestations in known tuberculosis or the initial clarification of symptoms in endemic areas. Ultrasound is also available in economically disadvantaged tuberculosis endemic areas. Ultrasound is therefore useful for TB screening in symptomatic individuals in high incidence areas, particularly for the clarification of extrapulmonary symptoms in individuals with known TB, and the monitoring of treatment responses. EUS and EUS-guided sampling may thus facilitate diagnosis in selected cases. With our comprehensive description of the appearance of extrapulmonary tuberculosis using abdominal and endoscopic ultrasound, including Color Doppler Imaging and CEUS, we hope to make medical colleagues more confident in recognizing and diagnosing the disease. As a result of national emergencies, there are significant migration movements in the world today. Physicians in countries with low rates of tuberculosis should consider this diagnosis in patients migrating from endemic areas.

## Figures and Tables

**Table 1:** Morphological changes of tuberculosis and corresponding findings on B-mode and contrast enhanced ultrasound.

Morphological Changes	Corresponding Findings in B-Mode Ultrasound	Correspondent Findings in Contrast-Enhanced Ultrasound
Granulomatous inflammation	Hypoechoic, ill-defined lesions	Hyperenhanced lesions
Granulomas	Hypoechoic round lesions	Hyperenhanced lesions
Caseous necrosis	Hypoechoic, heterogeneous, anechoic lesions	Hypo- or nonenhanced, heterogeneously enhanced lesions, contrast enhanced septations, contrast enhanced rim
Caseous abscess	Anechoic lesions, septations, heterogeneous masses, hyperechoic rim	Nonenhanced lesions, contrast enhanced rim, contrast enhanced septations
Fibrosis, strictures	leads to dilation of upstream fluid-filled structures (Bile ducts, Hydronephrosis, Caliectasia, hydrosalpinx)	The dilated structures are nonenhanced
Septations	Echoic fibrin strands in pleural effusion and ascites	Fibrin strands in pleura effusion and ascites are nonenhanced; septations in abscesses and lymph nodes may be enhanced
Calcifications	Point-shaped, crescent-shaped, ring-shaped hyperechoic reflexes with dorsal sound cancellation; for example, miliary/diffuse distribution, micro- and macrocalcifications in lesions, triangular lobar border in the kidney	No additional information
Fistulas	Hypoechoic or anechoic tubular structures between hypoechoic or anechoic caseous abscesses and surrounding structures; air reflexes with reverberation may occur upon contact with the gastrointestinal tract	Nonenhanced tubular structures

**Table 2:** Typical ultrasound features of extrapulmonary tuberculosis.

Organ	Tuberculosis Manifestations on Ultrasound
Lung	Subpleural hypoechoic nodules and lung consolidations
Pleura	Pleural effusion, fibrin strands, septations, pleural thickening and calcifications
Peritoneum, mesentery, omentum	Ascites, fibrin strands, septations, thickened peritoneum and mesentery, local encapsulated ascites Nodular lesions in the mesentery and adhesions
Lymph nodes	Enlarged, round, hypoechoic lymph nodes with hypo- or anechoic central areas
Liver	Hepatomegaly, multiple miliary hypoechoic lesions, macronodular hypoechoic lesions, hypoechoic tuberculomas, hypo- or anechoic caseous abscesses with hyperechoic rim, calcifications Serohepatic form with a thickened liver capsule and subcapsular nodular lesions
Spleen	Splenomegaly, multiple micronodular hypoechoic lesions, hypoechoic macronodular lesions.
Kidney	Caliectasia, hydrocalicosis, hydronephrosis, calcifications, lobar calcification, narrowed parenchymal margin, shrunken kidney
Pancreas	Diffuse hypoechoic enlargement, solitary or multiple hypoechoic lesions, peripancreatic lymphadenopathy
Gastrointestinal tract	Hypoechoic, heterogeneous wall thickening with loss of normal stratification, adjacent enlarged lymph nodes, fistulas
Pericardium	Pericardial effusion, fibrin strands, pericardial thickening, calcifications
